# Fbxo22 promotes cervical cancer progression via targeting p57^Kip2^ for ubiquitination and degradation

**DOI:** 10.1038/s41419-022-05248-z

**Published:** 2022-09-20

**Authors:** Min Lin, Jianan Zhang, Hakim Bouamar, Zhiwei Wang, Lu-Zhe Sun, Xueqiong Zhu

**Affiliations:** 1 Center for Uterine Cancer Diagnosis & Therapy Research of Zhejiang Province, Department of Obstetrics and Gynecology, The Second Affiliated Hospital of Wenzhou Medical University, Wenzhou, Zhejiang 325027 China; 2 Department of Cell Systems & Anatomy, School of Medicine, University of Texas Health Science Center at San Antonio, San Antonio, TX USA

**Keywords:** Cervical cancer, Cervical cancer

## Abstract

F-box only protein 22 (FBXO22) is a key subunit of the Skp1-Cullin 1-F-box protein (SCF) E3 ubiquitin ligase complex. Little is known regarding its biological function and underlying molecular mechanisms in regulating cervical cancer (CC) progression. In this study, we aim to explore the role and mechanism of FBXO22 in CC progression. The correlation between FBXO22 and clinicopathological characteristics of CC was analyzed by tissue microarray. MTT, colony formation, flow cytometry, Western blotting, qRT-PCR, protein half-life, co-immunoprecipitation, ubiquitination, and xenograft experiments were performed to assess the functions of FBXO22 and potential molecular mechanisms of FBXO22-mediated malignant progression in CC. The expression of FBXO22 protein in CC tissues was higher than that in adjacent non-tumor cervical tissues. Notably, high expression of FBXO22 was significantly associated with high histology grades, positive lymph node metastasis, and poor outcomes in CC patients. Functionally, ectopic expression of FBXO22 promoted cell viability in vitro and induced tumor growth in vivo, while knockdown of FBXO22 exhibited opposite effects. In addition, overexpression of FBXO22 promoted G1/S phase progression and inhibited apoptosis in CC cells. Mechanistically, FBXO22 physically interacted with the cyclin-dependent kinase inhibitor p57^Kip2^ and subsequently mediated its ubiquitination and proteasomal degradation leading to tumor progression. FBXO22 protein level was found negatively associated with p57^Kip2^ protein levels in patient CC samples. FBXO22 promotes CC progression partly through regulating the ubiquitination and proteasomal degradation of p57^Kip2^. Our study indicates that FBXO22 might be a novel prognostic biomarker and therapeutic target for CC.

## Introduction

Cervical cancer (CC) is one of the leading causes of cancer-related deaths in women worldwide, ranking in the fourth in overall morbidity and mortality among female malignancies with serious consequences in women’s life and health [1]. According to the latest published global cancer statistics covering 36 cancers in 185 countries, there are about 604,000 new cases of CC and ~342,000 deaths due to CC in 2020, with more than 85% of these deaths occurring in less developed regions [1]. With the promotion of the human papillomavirus (HPV) vaccine and the widespread availability of CC screening, the incidence and mortality of CC are slowly decreasing over the past 30 years in high-income countries, but in many developing countries, CC remains the second most common cancer and the leading cause of cancer death in women [1–3]. Cervical carcinogenesis is a multistep process involving viral infection, inactivation of tumor suppressor genes, activation of proto oncogenes, and immune escape. However, the mechanisms of CC initiation and progression have not been fully explored. It is of great significance to further investigate the detailed mechanisms of cervical carcinogenesis for the identification of potential targets for the diagnosis and treatment of CC.

The ubiquitin proteolysis system (UPS) regulates protein degradation via a protein post-translational modification mechanism called ubiquitination [4, 5]. The UPS is involved in the degradation of 80% of all intracellular proteins affecting genomic stability and signaling pathways in order to regulate cellular functions including cell proliferation and apoptosis [6–8]. Accumulated evidence indicates that F-box proteins are involved in tumor initiation, progression, metastasis, and drug resistance in human malignant tumors [9–11]. FBXO22, namely F-box protein 22 or F-box only protein 22, is one of the F-box protein (FBP) subfamily members, which are essential subunits of SCF E3 ubiquitin ligases in the UPS [12, 13]. FBXO22 increased hepatocellular carcinoma progression via targeting KLF4 and p21 for degradation [14, 15]. Knockdown of FBXO22 attenuated cell migration, invasion, and angiogenesis via regulation of the HIF-1α/VEGF pathway in melanoma [16].

On the other hand, FBXO22 plays a dual role in breast cancer and lung adenocarcinoma [17–19]. FBXO22 promoted breast cancer cell growth but inhibited cell metastasis [17]. Similarly, FBXO22 promoted lung cancer cell growth through the LKB1/AMPK/mTOR signaling pathway, but inhibited metastasis by suppressing Bach1 [18, 19]. FBXO22 blocked metastasis via suppression of MMP9-mediated migratory and invasive ability and VEGF-involved angiogenesis in human renal cell carcinoma (RCC) [20]. Currently, it is not clear whether FBXO22 also exerts these paradoxical functions in gynecological malignancies. Therefore, we carried out the current study to investigate biological functions of FBXO22 in cervical cancer and its underlying regulatory mechanism.

## Results

### FBXO22 expression is upregulated in CC tissues and correlated with neoplastic progression

**Fig. 1:**
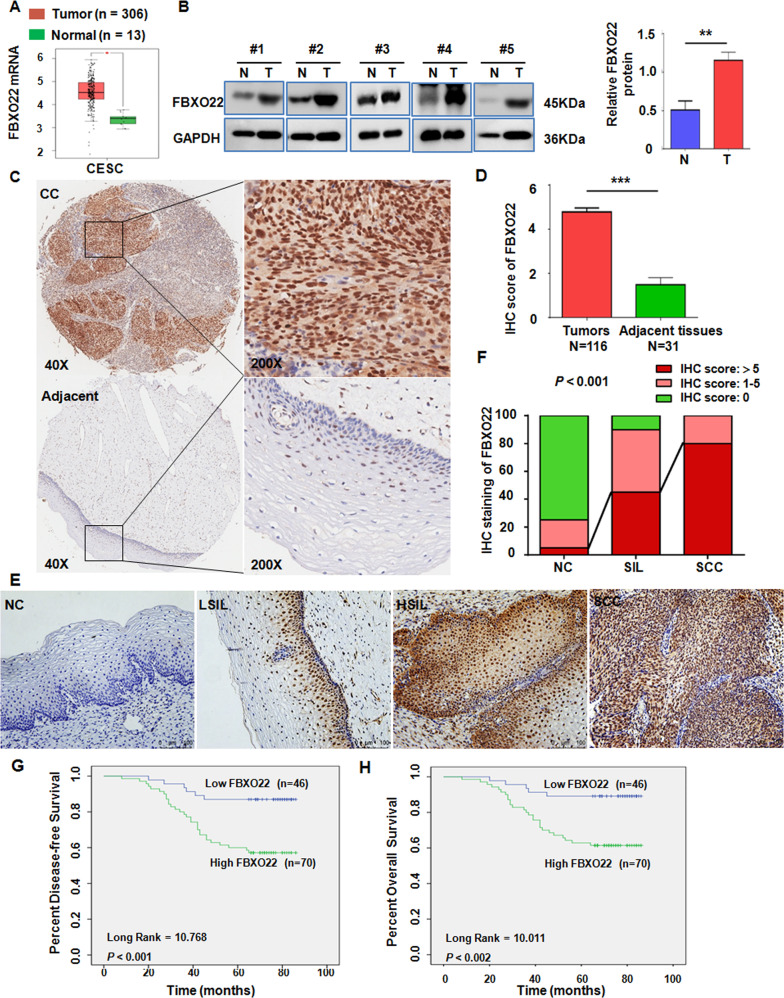
FBXO22 expression is upregulated in cervical cancer and correlated with neoplastic progression and survival of CC patients. **A** The expression of FBXO22 mRNA in human cervical cancers and corresponding normal cervical tissues from GEPIA database. CESC: Cervical squamous cell carcinoma and endocervical adenocarcinoma. *P < 0.05. **B** Representative images of Western blotting analysis of the FBXO22 protein level in tumor-adjacent cervical tissues (N) and cervical cancer tissues (T) of 5 CC patients (left panel) and the relative expression of FBXO22 protein is presented as normalized band density to the corresponding GAPDH band density (right panel). **P < 0.01. Data are expressed as mean ± SEM (*N* = 5). **C** Representative images of IHC staining with anti-FBXO22 antibody from cervical cancer (CC) tissues and adjacent cervical epithelial tissues in a human CC tissue microarray (×40 and ×200, magnification). **D** Scores of IHC staining are presented. ***P < 0.001. Data are expressed as mean ± SEM. **E** Representative images of IHC staining with anti-FBXO22 antibody in human normal cervical tissues (NC), low-grade squamous intraepithelial lesion (LSIL), high-grade squamous intraepithelial lesion (HSIL), and squamous cell carcinoma (SCC) (×400, magnification). **F** Histogram of FBXO22 protein expression in NC, squamous intraepithelial lesion (SIL), and SCC. **G**, **H** Kaplan–Meier analysis of **G** disease-free survival (DFS) and **H** overall survival (OS) of cervical cancer patients related to the levels of FBXO22 protein.

To explore the role of FBXO22 in CC, the expression of FBXO22 in the clinical samples was measured. *FBXO22* mRNA level was higher in CC tissues than in normal cervical tissues in the GEPIA database (http://gepia.cancer-pku.cn/index.html), which includes data from the TCGA and GTEx databases (Fig. 1A). Overexpression of FBXO22 was observed in CC tissues compared with adjacent cervical epithelial tissues by Western blotting assay in 5 fresh clinical CC and adjacent tissue specimens (Fig. 1B). Moreover, a CC tissue microarray, containing 116 cervical carcinomas and 31 adjacent cervical epithelial tissues, was performed to evaluate the difference of FBXO22 protein expression by IHC staining assay. As presented in Fig. 1C and D, the expression of FBXO22 in human CC tissues was higher than that in adjacent non-tumor tissues. The frequency of highly stained (IHC score > 5) CC samples was 60.34% (70/116), while it was only 6.45% (2/31) in the adjacent cervical tissues. Taken together, these results demonstrated that FBXO22 expression is elevated in human CC tissues.

To further investigate the relationship between FBXO22 and cervical neoplastic progression, formalin-fixed paraffin-embedded cervical tissues, including 20 normal cervical epithelium (NC), 20 cervical squamous intraepithelial lesion (SIL), and 20 cervical squamous cell cancer (SCC), were utilized to analyze the expression of FBXO22 protein by IHC analysis. Our data showed that the expression of FBXO22 protein not only in SCC tissues but also in SIL tissues was dramatically upregulated compared with the NC epithelium (Fig. 1E and F). Moreover, FBXO22 expression was also higher in SCC samples than that in SIL tissues (Fig. 1E and F). These data demonstrate the association of FBXO22 expression with the neoplastic progression of cervical epithelium.

### FBXO22 is associated with the poor prognosis of patients with CC

To determine the correlation between FBXO22 expression and clinicopathology of patients with CC, the IHC data of tissue microarray were further analyzed. As shown in Supplementary Table 1, the high expression of FBXO22 protein levels was significantly correlated with advanced histology grades and positive lymph node metastasis. However, none of age, pathologic type, TNM stage, and HPV infective status was statistically associated with FBXO22 protein expression (Supplementary Table 1). The multivariate Cox regression analysis revealed that high expression of FBXO22 was an independent risk factor for prognosis of OS and DFS in patients with CC (Supplementary Tables 2 and 3). Patients with high FBXO22 expression implicated a 4.135-folds and a 4.448 greater risk for recurrence and death, respectively (Supplementary Tables 2 and 3). Kaplan–Meier survival analysis illustrated that patients with high expression of FBXO22 have significantly shorter DFS (long rank = 10.768, *P* < 0.001) and OS (long rank = 10.011, *P* < 0.002) than those with low FBXO22 expression (Fig. 1G and H). Collectively, these data verified that CC patients with an increase in FBXO22 levels have a higher recurrence and mortality rate compared with those with the low FBXO22 expression. The expression of FBXO22 is negatively associated with the better prognosis of patients with CC, indicating that FBXO22 expression may be a novel prognostic biomarker for CC patients.

### Silencing FBXO22 inhibits cell proliferation and G1/S phase progression, and induces apoptosis

**Fig. 2:**
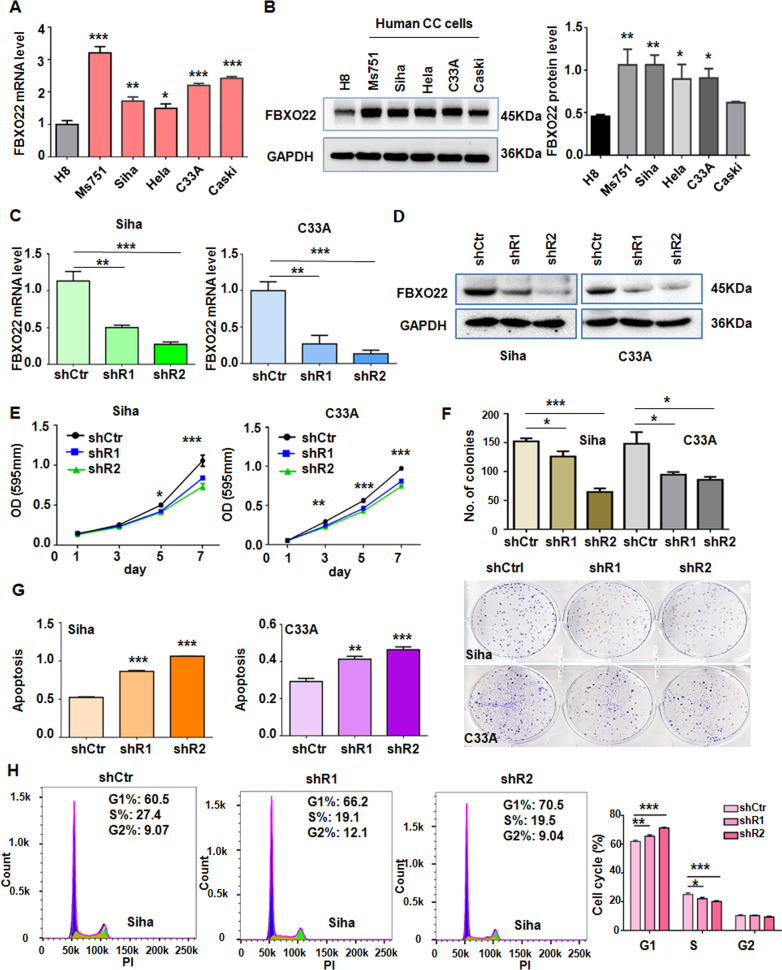
Knockdown of FBXO22 inhibits cell growth and G1/S phase progression, and induces apoptosis. **A**, **B** The relative mRNA (**A**) and protein levels (**B**) of FBXO22 in one human immortalized cervical cell line (H8) and five various human CC cell lines by qRT-PCR and Western blotting method, respectively. The FBXO22 protein level was presented as the intensity of FBXO22 band normalized to that of the corresponding GAPDH band of each CC cell line (**B**, right panel). *P < 0.05, **P < 0.01, ***P < 0.001. Data are shown as mean ± SEM from triplicate measurements. **C**, **D** The mRNA level (**C**) and protein level (**D**) of FBXO22 were detected after stable expression of FBXO22 shRNAs (shR1 and shR2) or control shRNA (shCtr) in SiHa and C33A cells. GAPDH level was used as a loading control. **P < 0.01, ***P < 0.001. Data are shown as mean ± SEM from triplicate measurements. **E**, **F** FBXO22 knockdown inhibited cells viability (**E**) and colony formation (**F**) in SiHa and C33A cell lines. *P < 0.05, ***P < 0.001. Data are shown as mean ± SEM from triplicate measurements. **G**, **H** Knockdown of FBXO22 induced the apoptosis (**G**) in SiHa and C33A cells and inhibited the G1/S phase cell cycle progression (**H**) in SiHa cells. Each data in the plots represents the means ± SEM from triplicate measurements. *P < 0.05, **P < 0.01, ***P < 0.001.

The expression of FBXO22 in five CC cell lines and one immortalized cervical cell line (H8) was detected by qRT-PCR and Western blotting assay. Our data showed a higher expression of FBXO22 in CC cell lines compared with the H8 cells (Fig. 2A and B). Clinically, the vast majority of CC patients are squamous cell carcinomas, accounting for more than 80%. As such, we selected squamous cell lines SiHa and C33A for the subsequent experiments to uncover the biological functions of FBXO22 in CC cells. FBXO22 was stably knocked down by lentiviral infection with two lentivectors expressing two different FBXO22 shRNAs (shR1 and shR2) in SiHa and C33A cell lines. The empty vector (shCtr) was used as control. The results from qRT-PCR and Western blotting revealed that the expression of either shR1 or shR2 reduced the mRNA and protein levels of FBXO22 (Fig. 2C and D). Cell viability was decreased in SiHa and C33A cells after FBXO22 depletion by MTT assay (Fig. 2E). Consistently, colony formation assay data confirmed that silencing FBXO22 expression reduced the colony formation of SiHa and C33A cells (Fig. 2F). Since cell cycle and apoptosis are the key links to cell growth, we further detected the effects of FBXO22 on the cell cycle progression and apoptosis in SiHa and C33A cells. Knockdown of FBXO22 induced more cells arrested at G1 phase (Fig. 2H and Supplementary Fig. 1). Moreover, knockdown of FBXO22 also induced apoptosis of SiHa and C33A cells (Fig. 2G). In conclusion, these results indicate that suppression of FBXO22 represses cell growth in part due to promotion of cell cycle arrest and apoptosis.

### Overexpression of FBXO22 promotes proliferation and inhibits apoptosis

**Fig. 3:**
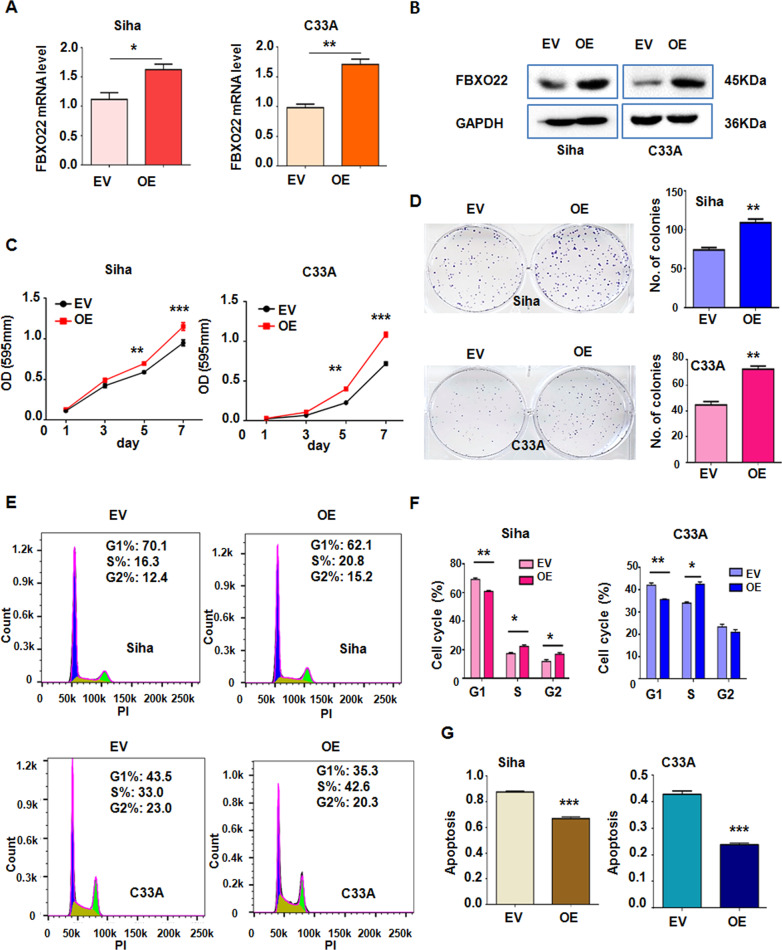
Ectopic expression of FBXO22 promotes cell growth and G1/S phase progression, and inhibits cell apoptosis. **A**, **B** The mRNA levels (**A**) and protein levels (**B**) of FBXO22 were detected in FBXO22 overexpressing (OE) or empty vector (EV)-transfected SiHa and C33A cells. GAPDH level was used as a loading control. FBXO22 mRNA level in panel **A** is the means ± SEM from triplicate measurements. *P < 0.05, **P < 0.01. **C**, **D** Overexpressed FBXO22 promoted viability (**C**) and colony formation (**D**) of SiHa and C33A cells. Each data point is means ± SEM from triplicate measurements. **P < 0.01, ***P < 0.001. **E**, **F** Overexpression of FBXO22 accelerated G1 to S phase cell cycle progression. Each data point is means ± SEM from triplicate measurements. *P < 0.05, **P < 0.01. **G** Overexpression of FBXO22 suppressed the apoptosis of SiHa and C33A cells. Each data in the plots are presented as the means ± SEM from triplicate measurements. ***P < 0.001.

To confirm that FBXO22 impacts on cell growth, SiHa and C33 A cells with stable overexpression of FBXO22 (OE group) were established. The data from qRT-PCR and Western blotting demonstrated that FBXO22 overexpressing SiHa and C33A cells had an elevated mRNA and protein levels in comparison to their respective control cell (Fig. 3A and B). In both CC cell lines, ectopic overexpression of FBXO22 enhanced the cell viability and colony formation compared with the control group (Fig. 3C and D). In addition, overexpression of FBXO22 increased the cell numbers in S phase compared with control group (Fig. 3E and F). Overexpression of FBXO22 also significantly inhibited the apoptosis of SiHa and C33A cells compared to the control group (Fig. 3G). These data further demonstrate that FBXO22 promotes the growth and survival of the CC cells in vitro. Interestingly, our migration and invasion data showed that overexpression of FBXO22 inhibited cell migratory and invasive abilities in cervical cancer (Supplementary Figs. 3 and 4).

### FBXO22 promotes tumor growth in vivo

**Fig. 4:**
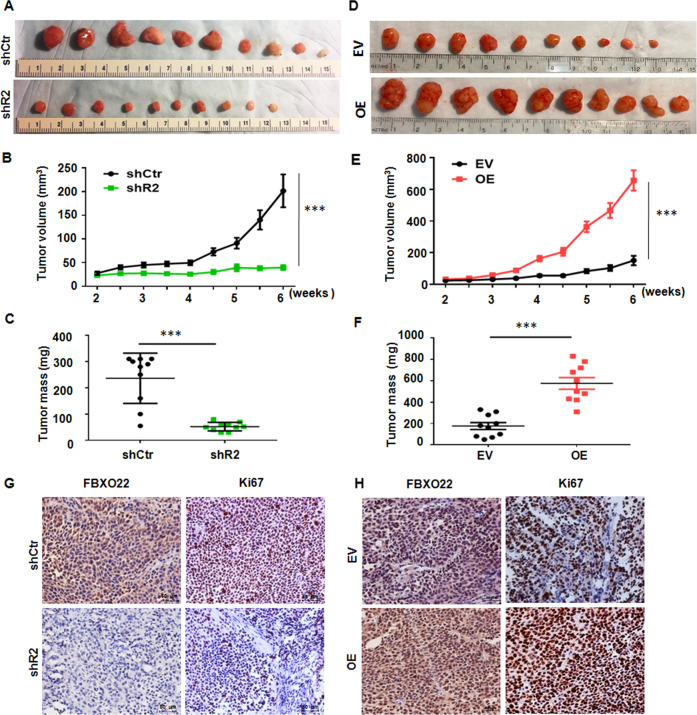
FBXO22 promotes tumor growth in vivo. **A** Female nude mice were inoculated subcutaneously with SiHa control cells (shCtr) FBXO22 knockdown cells (shR2) (2 × 10^6^ cells per mouse and the experiment lasted 6 weeks). The photographs of dissected tumors are presented. **B**, **C** Tumor growth curve (**B**) and terminal tumor weights (**C**) in the mice are presented as means ± SEM (*N* = 10). ***P < 0.001. **D** Female nude mice were inoculated subcutaneously with SiHa control cells (EV) and FBXO22 overexpressing cells (OE) (2 × 10^6^ cells per mouse and the experiment lasted 6 weeks). The photographs of dissected tumors are presented. **E**, **F** Tumor growth curve (**E**) and terminal tumor weights (**F**) in the mice are presented as means ± SEM (*N* = 10). ***P < 0.001. **G**, **H** Representative images of IHC staining with anti-FBXO22 and anti-Ki67 antibodies in tumor tissues (×200, magnification). shCtr: control shRNA. shR2: FBXO22 knockdown with shR2. EV: empty vector, OE: FBXO22 overexpression.

To further explore the effects of FBXO22 on tumorigenesis in vivo, SiHa cells with knockdown or overexpression of FBXO22 were subcutaneously injected into the dorsal root of right thighs in five-week-old BALB/c female nude mice. The average tumor volumes and weights in the FBXO22 knockdown group were markedly decreased compared to the control group (Fig. 4A–C). Conversely, overexpression of FBXO22 resulted in an increased tumor growth rate and terminal tumor weight (Fig. 4D–F). IHC staining of the paraffin embedding mouse tumors showed in Fig. 4G and H that the expression of FBXO22 protein was reduced in FBXO22 knockdown group, while FBXO22 expression was increased in FBXO22 overexpression group in comparison to the control group. The number of Ki67 positive proliferating cells was decreased in the FBXO22 knockdown group, while it was increased in the FBXO22 overexpression group. We also measured the expression of E-cadherin by IHC in these tumors. We found that FBXO22 overexpression increased the expression of E-cadherin, while FBXO22 depletion decreased the expression of E-cadherin in xerographs tumors (Supplementary Fig. 5). Therefore, these data demonstrated the oncogenic role of FBXO22 in CC.

### FBXO22 regulates p57^Kip2^ protein level and physically interacts with p57^Kip2^

**Fig. 5:**
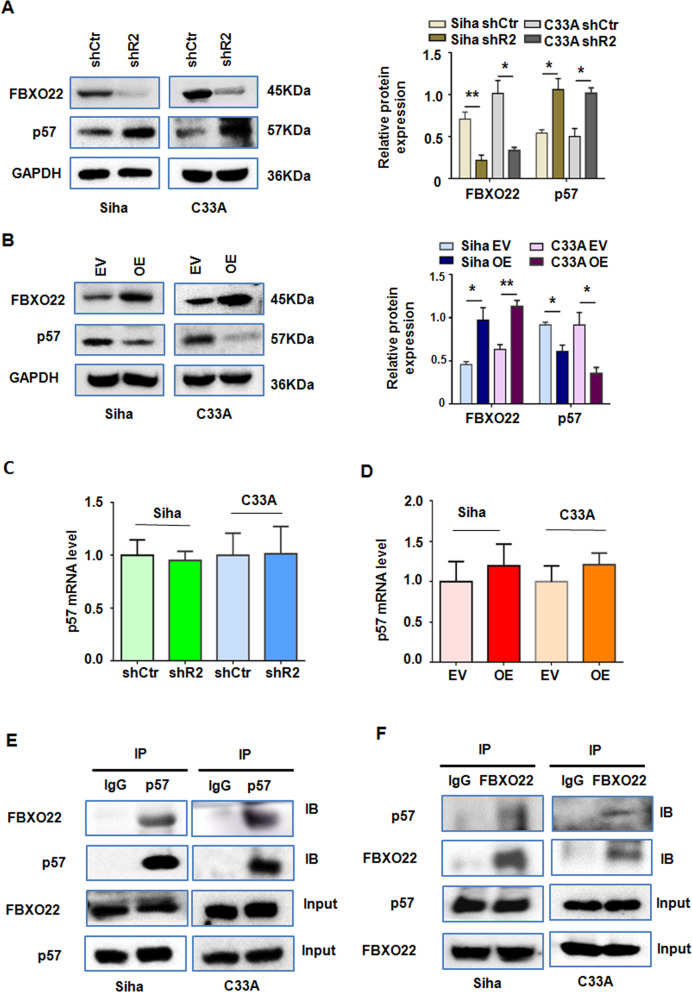
FBXO22 regulates p57Kip2 protein level and physically interacts with p57Kip2. **A**, **B** *FBXO22* knockdown increased p57 protein expression (**A**) and its ectopic expression reduced p57Kip2 protein expression (**B**) in SiHa and C33A cells detected by Western blotting analysis. Relative FBXO22 and p57Kip2 protein level is presented as normalized band density to the corresponding GAPDH band density. Each data in the plots is the mean ± SEM from three independent Western blots. *P < 0.05, **P < 0.01. shCtr: control shRNA, shR2: shFBXO22-2, EV: empty vector, OE: FBXO22 overexpression. **C**, **D** *FBXO22* knockdown (**C**) or overexpression (**D**) showed no effect on the p57Kip2 mRNA expression in SiHa and C33A cells. Each data in the plots are expressed as the means ± SEM from triplicate measurements. **E**, **F** FBXO22 and p57Kip2 proteins physically interact with each other in SiHa and C33A cells by IP assays as detected by immunoblotting (IB) for FBXO22 in the precipitated proteins by an anti-p57Kip2 antibody (**E**) and for p57Kip2 in the precipitated proteins by an anti-FBXO22 antibody (**F**).

To determine the mechanism of FBXO22-mediated cell growth and CC progression, the expression of several proteins in CC cells including those that regulate cell cycle was measured by Western blotting experiments in CC control and FBXO22 knockdown cells. We found that FBXO22 reduced the expression of cyclin-dependent kinase inhibitor p57^Kip2^ coded by *CDKN1C*. As presented in Fig. 5A, the expression of p57^Kip2^ protein was obviously increased in CC cells with FBXO22 knockdown. Conversely, ectopic expression of FBXO22 diminished the p57 protein levels compared to the empty vector group in CC cells (Fig. 5B). We also observed that FBXO22 expression had a certain negative correlation trend with p57 in multiple CC cell lines (Supplementary Fig. 6A, B). On the other hand, neither downregulation nor upregulation of FBXO22 had an effect on the expression of p57^Kip2^ mRNA level in SiHa and C33A cells (Fig. 5C, D), indicating that FBXO22 might affect the stability of p57^Kip2^ protein through post-translational regulation. Moreover, our IP assay revealed that an anti-p57^Kip2^ antibody was able to pull down FBXO22 protein, while an anti-FBXO22 antibody pulled down p57^Kip2^ in both SiHa and C33A cells, suggesting that FBXO22 physically interacts with p57^Kip2^ in CC cells (Fig. 5E and F). FBXO22 includes 1–403 amino acids and contains an N-terminal F-box domain and a C-terminal domain. We have constructed a FBXO22 mutant (Flag-FBXO22Δ) that was deleted 101–403 amino acids, and then did the IP experiment in 293T cell. 293T cells were transfected with myc-p57, Flag-FBXO22, and Flag-FBXO22Δ. The result showed that anti-Flag antibody was able to pull down myc protein in Flag-FBXO22 WT, but not in the FBXO22Δ (Supplementary Fig. 7A).

### FBXO22 regulates p57^Kip2^ stability via ubiquitination/proteasome degradation

**Fig. 6:**
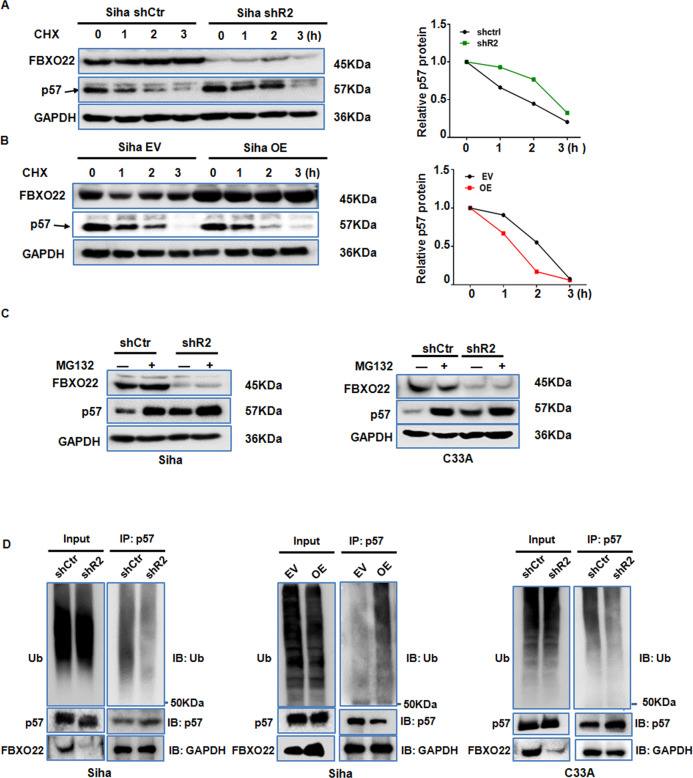
FBXO22 regulates the ubiquitination and degradation of p57Kip2 protein. **A**, **B** SiHa cells with FBXO22 knockdown (**A**) or overexpression (**B**) were treated with cycloheximide (CHX) at 100 μM, and then harvested at the indicated time points. The cell lysate was analyzed by the Western blotting for FBXO22, p57Kip2, and GAPDH levels (left panel). Quantification of the p57 levels relative to GAPDH expression is also shown (right panel). **C** Western blotting was used to detect the changes of p57Kip2 protein in SiHa and C33A cells without or with FBXO22 knockdown before and after MG132 (20 μM) treatment. **D** The levels of ubiquitinated p57Kip2 was measured in immunoprecipitated p57Kip2 in the CC cells with FBXO22 knockdown or overexpression.

Given the fact that FBXO22 is an integral part of the SCF E3 ubiquitin ligase complex, which regulates protein stability, we next measured the stability of p57^Kip2^ protein in CC cells with stable knockdown or overexpression of FBXO22. CC cells were treated with 100 μM cycloheximide (CHX) to block protein synthesis and the protein levels of p57^Kip2^ was measured with Western blotting after hourly treatment with CHX during three hours. We observed a slower decrease of p57^Kip2^ protein levels in SiHa cells with FBXO22 knockdown and a faster decrease in SiHa cells with FBXO22 overexpression in comparison to their respective control cells (Fig. 6A and B) indicating that FBXO22 decreases p57^Kip2^ half-life. Consistently, blockade of protein degradation with the proteasome inhibitor MG132 was equally effective in stabilizing p57^Kip2^ as the knockdown of FBXO22 in both SiHa and C33A cells (Fig. 6C) indicating that p57^Kip2^ level is controlled by ubiquitination and proteasomal degradation. To verify whether FBXO22 regulates p57^Kip2^ ubiquitination, an ubiquitin-expressing vector was transiently transfected into the FBXO22 knockdown or overexpressing cells. We found that p57^Kip2^ ubiquitination was decreased in the SiHa and C33A cell lines with FBXO22 knockdown, while it was increased in SiHa cells after FBXO22 overexpression (Fig. 6D). Subsequently, 293T cells were transfected with myc-p57, HA-ubiquitin, Flag-FBXO22, and Flag-FBXO22Δ. It showed an increase of polyubiquitinated p57 protein in the cells transfected with wild-type FBXO22, as well as a decrease in the cells transfected with FBXO22Δ mutant (Supplement Fig. 7B). Altogether, FBXO22 can promote p57^Kip2^ ubiquitination and degradation in CC cells.

### FBXO22 promotes HCC cell growth and survival by silencing p57^Kip2^ expression

**Fig. 7:**
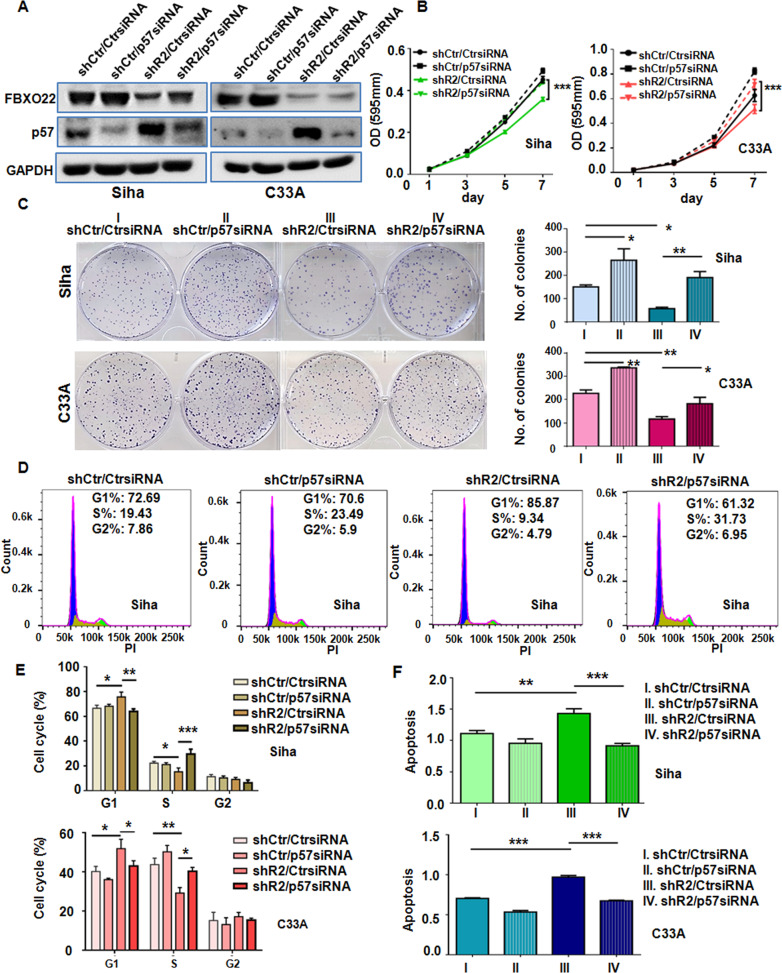
FBXO22 promotes HCC cell growth and survival by silencing p57Kip2 expression. **A** The protein levels of FBXO22 and p57Kip2 were detected by Western blotting in SiHa and C33A control (shCtr) and FBXO22 knockdown (shR2) cells transfected with a control siRNA (CtrsiRNA) or a p57Kip2 siRNA (p57siRNA). **B**, **C** MTT (**B**) and colony formation (**C**) assays were used to measure the viability and proliferation in SiHa and C33A cells with or without FBXO22 and/or p57 knockdown. **D**, **E** Cell cycle was analyzed in SiHa and C33A cells with or without FBXO22 and/or p57 knockdown. **F** Cell apoptosis was analyzed in SiHa and C33A cells with or without FBXO22 and/or p57 knockdown. Data are shown as mean ± SEM from triplicate measurements. *P < 0.05, **P < 0.01, ***P < 0.001.

To determine whether the regulation of p57^Kip2^ expression is a major mechanism by which FBXO22 exerts its malignancy-promoting activity, we knocked down p57^Kip2^ in the SiHa and C33A cells to investigate whether FBXO22 depletion-induced increase of p57^Kip2^ protein level was responsible for the reduced cell growth and increased apoptosis in FBXO22 knockdown cells. The knockdown of p57^Kip2^ by the transfection of an siRNA was verified by Western blotting (Fig. 7A). MTT and colony formation assays revealed that p57^Kip2^ knockdown abrogated the reduced cell viability and colony formation in SiHa and C33A cells with FBXO22 knockdown (Fig. 7B and C). Moreover, downregulation of p57^Kip2^ abolished cell cycle arrest at G1 phase induced by FBXO22 depletion in SiHa and C33A cells (Fig. 7D, E and Supplementary Fig. 2). Furthermore, p57^Kip2^ siRNA transfection rescued the apoptosis of SiHa and C33A cells caused by FBXO22 downregulation (Fig. 7F). Notably, the cells transfected with FBXO22Δ mutant did not increase the colony formation (Supplementary Fig. 7C, D). Strikingly, we also observed that FBXO22 can regulate the expression of PDL1, Snail, p-p53, and KDM4B in CC cells (Supplementary Fig. 8). Thus, FBXO22 exerts its oncogenic role in part by targeting p57^Kip2^ protein stability.

### FBXO22 is negatively associated with p57^Kip2^ expression in xenograft and clinical tumor tissues

**Fig. 8:**
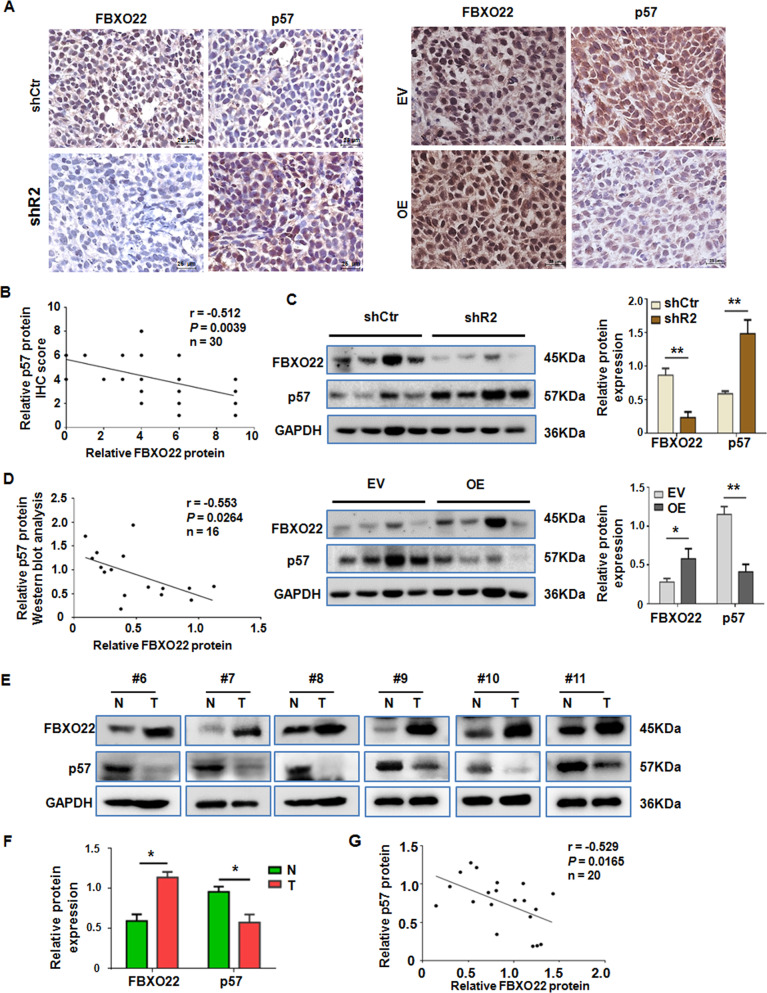
FBXO22 is negatively associated with p57Kip2 expression in xenograft and clinical tumor tissues. **A** Representative images of IHC staining with anti-FBXO22 and anti-p57Kip2 antibodies in tissue sections of the tumors formed by the control, FBXO22 knockdown (shR2), or overexpressing (OE) SiHa cells (×400, magnification). **B** Linear correlation analysis of FBXO22 and p57Kip2 IHC scores in mouse xenograft tissues. **C** Western blotting analysis of the FBXO22 and p57Kip2 protein levels in the tumors formed by the control, FBXO22 knockdown (shR2), or overexpressing (OE) SiHa cells. The relative FBXO22 and p57Kip2 protein level is presented as normalized band density to the corresponding GAPDH band density. Each data in the plots is the mean ± SEM from four randomly selected tumors. *P < 0.05, **P < 0.01. **D** Linear correlation analysis of the relative FBXO22 and p57Kip2 protein levels in the selected mouse tumors formed by the control, FBXO22 knockdown (shR2), or overexpressing (OE) SiHa cells. **E** Representative images of Western blotting analysis of the FBXO22 and p57Kip2 protein levels in tumor-adjacent cervical tissues (N) and cervical cancer tissues (T) of 6 CC patients. **F** Relative expression of FBXO22 and p57Kip2 protein as normalized band density to the GAPDH band density. Each data in the plot is the mean ± SEM from 10 normal or tumor tissue samples. *P < 0.05. **G** Linear correlation analysis of the relative FBXO22 and p57 protein levels in 20 tissue samples.

The expression of FBXO22 and p57^Kip2^ in the mouse xenograft tumor tissues was analyzed by IHC. The results showed that the expression of p57^Kip2^ protein was inversely associated with FBXO22 protein levels in the xenograft tumors formed by the FBXO22 knockdown cells (Fig. 8A left panel) and by the FBXO22 overexpressing cells (Fig. 8A right panel) in comparison their respective control cell-formed tumors. Consistently, linear correlation analysis of the IHC scores showed that the expression of FBXO22 and p57 proteins was negatively correlated (*r* = −0.512, *P* < 0.01) (Fig. 8B). We also confirmed this negative correlation between FBXO22 and p57^Kip2^ in the xenograft tumors by Western blotting for the two proteins in tumor tissue extracts (Fig. 8C). Linear correlation analysis of the relative band intensity of FBXO22 and p57^Kip2^ in the Western blots also showed the negative correlation between the expression of the two proteins (*r* = −0.553, *P* < 0.05) (Fig. 8D). We next investigated the association between FBXO22 and p57 expressions in clinical tissue samples. The expression of FBXO22 and p57^Kip2^ proteins in 10 paired cervical cancer tissues and adjacent non-tumor tissues were analyzed by Western blotting. The results unveiled that the significant upregulation of FBXO22 protein was associated with a significant reduction of p57^Kip2^ protein in CC tissues in comparison to their matched adjacent tissues (Fig. 8E and F). Thus, FBXO22 expression was negatively associated with p57 expression in CC tissues as shown in a linear regression analysis (*r* = −0.529, *P* < 0.05) (Fig. 8G).

## Discussion

Accumulating evidence indicates that FBXO22 plays an essential role in a broad range of human cancers [9]. However, the effects of FBXO22 on CC development and progression and underlying molecular insights are lacking. In the present study, we report for the first time that ectopic expression of FBXO22 promoted cell viability and inhibited apoptosis in CC cells via promoting the ubiquitination and proteasomal degradation of p57^Kip2^ uncovering a new mechanism by which FBXO22 promotes malignant properties of cervical cancer (Supplementary Fig. 9).

FBXO22 has been reported to be highly expressed and linked to poor prognosis in several human cancers. For example, increased expression of FBXO22 has been shown in HCC [14], melanoma [16], and lung cancer [18]. High expression of FBXO22 is correlated with worse prognosis in HCC [15] and lung cancer [18]. However, contrary to these reports, lower level of FBXO22 was linked to poor prognosis in breast cancer patients and renal cell carcinoma patients [20, 21]. Herein, we report that FBXO22 expression is upregulated in CC tissues and associated with higher histology grade and lymph node metastasis in CC patients. Higher expression of FBXO22 is associated with worse OS and DFS of CC patients, indicating that FBXO22 is an independent risk factor of the recurrence and prognosis of CC patients.

FBXO22 was shown to promote proliferation, migration, and invasion of HCC cells and facilitated their tumor growth in vivo [14]. Moreover, upregulation of FBXO22 enhanced proliferation and colony formation in breast cancer cells [17]. FBXO22 overexpression elevated cell growth in non-small cell lung cancer (NSCLC) [18]. Consistently, FBXO22 was also reported to enhance colorectal cancer development [22]. Moreover, FBXO22 increased migratory and invasive ability and angiogenesis in melanoma cells [16]. Paradoxically, overexpression of FBXO22 blocked EMT formation, cell migration and invasion, and metastasis in breast cancer, lung cancer, and RCC [17, 19–21]. In addition, FBXO22 increased cisplatin sensitivity of lung cancer cells [23]. Consistent with its tumor-promoting activity, our study demonstrated that FBXO22 enhanced cell viability, suppressed cell apoptosis, and increased xenograft tumor growth of CC. Further studies are needed to explore the effects of FBXO22 on migratory and invasive properties of CC cells.

FBXO22 have been shown to regulate the stability of various proteins including p21^Cip1^ [15], KLF4 [14], Snail [17], HDM2 [21], LKB1 [18], CD147 [23], Bach1 [19], and PTEN [22]. We identified p57^Kip2^ as a new substrate of FBXO22 in CC cells. It is well known that p57^Kip2^, a cyclin-dependent kinase inhibitor belonging to the Cip/Kip family, acts as a tumor suppressor via regulation of growth, cell cycle, and apoptosis [24–26]. Accumulating evidence has indicated that p57^Kip2^ protein is frequently found to be downregulated in cancers. One study showed that p57^Kip2^ expression was downregulated via Jab1/CSN5 pathway in liver cancer, leading to promotion of cell growth and invasion [27]. Another study revealed that p57^Kip2^ inhibited cell proliferation in gastric cancer [28]. In addition, lncRNA SNHG17 promoted gastric cancer progression by epigenetically silencing p57^Kip2^ and p15^INK4b^ [29]. Similarly, we found that p57^Kip2^ functioned as a tumor suppressor in CC cells via regulating apoptosis and cell cycle. Importantly, p57^Kip2^ was ubiquitinated and degraded by FBXO22 in CC cells. Our rescue experiments demonstrated that FBXO22 exerts its oncogenic functions in part via targeting p57 degradation in CC cells.

Collectively, we report the tumor-promoting role of FBXO22 in cervical carcinogenesis. This mechanism of FBXO22-mediated cervical tumor progression is apparently by targeting ubiquitination and degradation of p57^Kip2^. Our study provides an experimental basis for targeting FBXO22 as a potential strategy for cervical cancer intervention.

## Materials and methods

### Ethics statement

The human tissue study was approved by the ethical committee of the Second Affiliated Hospital of Wenzhou Medical University and conducted according to the Helsinki declaration. Informed consent was obtained from all subjects prior to participation in the study. Cervical tissue sections, including normal cervical, SIL and SCC, used for this study were cut from leftover tissue blocks from consented patients. A CC tissue microarray was purchased from Shanghai Outdo Biotech Company (Shanghai, China). The animal experiments were conducted following appropriate guidelines. They were approved by the Institutional Animal Care and Use Committee and monitored by the Department of Laboratory Animal Resources at the University of Texas Health Science Center at San Antonio.

### Cell lines and cell culture

Human cervical cancer cell lines, including SiHa, C33A, HeLa, CaSki, and MS751 cell lines, were obtained from the American Type Culture Collection (ATCC, Manassas, VA). The human cervical immortalized epithelial cell line H8 was purchased from the Tong Pai Biotechnology Co., LTD (Shanghai, China). These cell lines were all authenticated with short tandem repeat DNA markers provided by the ATCC which were last tested between 2018 and 2021. SiHa, HeLa and MS751 cells were maintained in DMEM medium (Invitrogen, Carlsbad, CA), and C33A, CaSki, and H8 cells were cultured in RPMI1640 medium. All cell cultures were supplemented with 10% fetal bovine serum (FBS), 100 U/mL penicillin, and 100 μg/mL streptomycin (Invitrogen, Carlsbad, CA) in a humidified incubator with 5% CO_2_ at 37 °C.

### Plasmids, lentiviral vectors, and stable cell lines

Two *FBXO22* shRNA (shR1 and shR2) expression vectors and the paired backbone vector pGIPZ Lentiviral shRNA (shCtr) were purchased from Shanghai Jiao Tong University (Shanghai, China). The sequence of *FBXO22* shR1 is 5′ TGC TGT TGA CAG TGA GCG CCC AGA GTA TTA CAT CAT CTT ATA GTG AAG CCA CAG ATG TAT AAG ATG ATG TAA TAC TAT GGT TGC CTA CTG CCT CGG A-3′ and that of FBXO22 shR2 is 5′-TGC TGT TGA CAG TGA GCG CGG CCA TAA GAG AGC AAG GAA ATA GTG AAG CCA CAG ATG TAT TTC CTT GCT CTC TTA TGG CCA TGC CTA CTG CCT CGG A-3′. *FBXO22* cDNA expression vector and the matched backbone vector EX-NEG-Lv203 were purchased from GeneCopoeia (Rockville, MD, USA). Each of these lentivectors with two packaging vectors (psPAX2 and pMD2.G) (Addgene, Inc.) into the HEK293T cells using Lipofectamine^®^ 2000 (Thermo Fisher Scientific, Inc.) for lentivirus production. Subsequently, CC cells including SiHa and C33A cells were infected by lentivirus to establish stable FBXO22 knockdown or overexpressing cells as previously described [30].

### Cell viability assay and colony formation assay

SiHa and C33A cells with stable FBXO22 knockdown or overexpression were cultured in a 96-well plate with 100 μL culture medium in each well. At the specified time (day 1, 3, 5, and 7), a 50 μL 3-(4,5-Dimethylthiazol-2-yl)-2,5-diphenylte-trazolium bromide solution (MTT, 2 mg/ml in PBS) was added to each well and incubated for 2 h. The supernatant was then discarded and 100 μL dimethyl sulfoxide (DMSO) (Sigma-Aldrich, MO, USA) was added to each well to dissolve the MTT product formazan. The plate was shaken at 250 rpm for 10 min, and the OD value at 592 nm was measured to calculate the cell viability. For colony formation assay, briefly, CC cells were seeded in a six-well plate and incubated for 10–14 days when noticeable colonies were formed. Cell colonies were fixed using 100% methanol for 10 min, stained with 0.1% crystal violet for 10 min at room temperature, and photographed.

### Quantitative real-time PCR assay

Total RNA (2 μg) extracted from various cell lines was reverse-transcribed into cDNA using random primers and M-MLV reverse transcriptase (Invitrogen Life Technology, Grand Island, NY). Quantitative real-time PCR was performed using Power SYBR Green PCR Mix (Life Technology Company). The sequences of primers were as follow: FBXO22: 5′-ATG GGA TCA GGT AGC AAT CGA C-3′ (forward) and 5′-CCA CAC GAA GTT CAG GGT TAT C-3′ (reverse). p57: 5′-GAT CAA GAA GCT GTC CGG GC-3′ (forward) and 5′-GAT CTC TTG CGC TTG GCG AAG-3′ (reverse). Actin beta: 5′-ACA GAG CCT CGC CTT TGC CGA T-3′ (forward) and 5′-GGC CTC GTC GCC CAC ATA GGA-3′ (reverse). The primers were all synthesized by Integrated DNA Technologies (IDT) Company (Coralville, IA, USA).

### Small interfering RNAs (siRNAs) targeting *CDKNC1* (p57^Kip2^)

A pool of four small interfering RNAs (siRNAs) targeting p57^Kip2^ and non-specific control (Ctr siRNA) were purchased from Thermo Fisher (Waltham, MA, USA) and transfected into the CC cells in the presence of Dharma (Thermo Fisher, USA). The pool sequences of p57 siRNA were as follow: CCG CUG GGA UUA CGA CUU C, GGC CUC UGA UCU CCG AUU U, GAG CCA AUU UAG AGC CCA A, CUG AGA AGU CGU CGG GCG A. A single p57^Kip2^ siRNA purchased from Sigma-Aldrich (MO, USA) was also used and the sequence was CAC UAG CUC GGU UAU UGG U.

### Flow cytometry for cell cycle analysis

CC cells were harvested with trypsin without EDTA and washed twice with pre-cooled 1× PBS. Then pre-cooled 70% ethanol at 4 °C was added to the cell pellet to fix the cells, and transferred to −20 °C overnight. The cells were washed once with PBS, stained with a solution containing 20 g/mL RNAase and 50 g/mL propidium iodide in dark at room temperature for 30 min, and then analyzed in a flow cytometer for cell cycle distribution. The experiment was repeated for 3 times.

### ELISA for detecting apoptosis

Cell apoptosis was measured with an apoptosis ELISA kit from Roche (catalog # 177425). Briefly, CC cells were harvested with trypsin digestion, and the cells were washed twice with 4 °C cold PBS. Then the cell pellet was collected and lysed on ice with the apoptosis lysis buffer for 30 min. The supernatant was collected after centrifugation at 1200 × *g* at 4 °C for 10 min. The Bio-Rad protein assay (Dye Reagent Concentrate #500-0006) was used to measure the protein concentrations in the supernatant according to the manufacturer’s protocol. The supernatant containing 6 μg protein was mixed with 120 μL immunoreaction reagent (including anti-DNA-POD, anti-histone biotin) and incubation buffer at 1:1:18 volume ration and added to a Streptavidin-coated 96-well plate and incubated on a shaker at 200 rpm for 2 h in the dark at room temperature. The solution was carefully removed and a 100 μL substrate buffer was added. The plate was incubated on a shaker at 200 rpm for a few min at dark at room temperature until color changes to dark green. Finally, the OD values at 405 nm and 490 nm were, respectively, detected and the apoptotic index was calculated. Each experiment was repeated three times.

### Migration and invasion assays

Migration and invasion assays were performed using the Transwell chambers (8.0 μm pore size with polycarbonate membranes) with (invasion) or without (migration) growth factor-reduced Matrigel ((BD Biosciences, NJ, USA). 4 × 10^4^ SiHa cells or 7 × 10^4^ C33A cells in the medium without the FBS were seeded in the upper chamber for the migration assay. In the same chamber, 6 × 10^4^ SiHa cells or 8 × 10^4^ C33A cells were plated into the FBS-free medium for the invasion assay. Growth medium containing 10% FBS was used as a chemoattractant in the lower chamber. After indicated times (16 h for SiHa cell and 20 h for C33A cell), migrated and invaded cells were fixed in the membrane using 90% methanol and stained with 0.1% crystal violet. The cotton cells in the upper chamber were removed gently. Cells were counted under an inverted microscope at ×200 magnification.

### Western blot analysis

SiHa and C33A cells were lysed in a lysis buffer (50 mM Tris-HCl pH 7.4, 150 mM NaCl, and 0.5% NP-40) containing protease and phosphatase inhibitors with a Mini tablet (Thermo Fisher, A32961) added to 10 mL of the lysis buffer. The expression of FBXO22, p57^Kip2^, and GAPDH was examined by Western blotting analysis as previously described [31]. The following primary antibodies were used for Western blotting analysis: mouse anti-FBXO22 (1:500, sc-100736, Santa Cruz), rabbit anti-p57 (1:1000, sc-1040, Santa Cruz), mouse anti-Ub (1:500, sc-166355, Santa Cruz), mouse anti-GAPDH (1:2500, 80602-840, Calbiochem) Image J software was used for the measurement of band density and relative expression of a protein the ratio of its band density normalized by that of its corresponding GAPDH protein band.

### Immunohistochemical staining (IHC)

The IHC staining assay was conducted as described previously [32]. Images were analyzed by two independent pathologists. Results were determined by the percentage of positive cells and staining intensity of nuclear under the microscope. The final positive nuclear staining cells were scored as followed: the percentage of positive cells (0–25% = 0, 26–50% = 1, 51–75% = 2, and >75% = 3), multiplied the staining intensity (no staining = 0, light yellow = 1, brown = 2, strong brown = 3). The final scores were divided into low expression (0–5) and high expression (>5). The rabbit anti-FBXO22 (13606-1-AP, Protein-tech), mouse anti-Ki67 (550609, Biosciences), and rabbit anti-p57^Kip2^ (sc-1040, Santa Cruz) antibodies were, respectively, diluted to 1:400, 1:100, and 1:200.

### Immunocytochemistry (ICC)

CC cells were fixed in 100% methanol for 5 min and permeabilized with PBS plus 0.3%Tween for 5 min. Then, PBS plus 0.025% Tween was used to wash the cells two times. Goat serum (10%) was used to block non-specific protein-protein interactions for 1 h at room temperature. The cells were then incubated with mouse anti-FBXO22 (1:400, sc-100736, Santa Cruz) and rabbit anti-p57^Kip2^ (1:100, sc-1040, Santa Cruz) antibodies at 4 °C overnight. After 3 times washes with PBS, the cells were incubated with an Alexa Fluor®-labeled secondary antibody (Alexa Fluor® 488 (green) anti-rabbit IgG or Alexa Fluor® 568 (red) anti-mouse IgG) at 1:2000 for 1 h at room temperature. The images were obtained using a fluorescence microscope (Olympus).

### Immunoprecipitation (IP) assay

CC cells were planted in a 10 cm culture dish with a density of about 50%. On the next day, the corresponding plasmid was transfected into cells. After 20 h, the cells were treated with 20 μM MG132 for another 6 h. The cells were harvested with trypsin, washed twice with PBS, and lysed on ice within the lysis buffer for Western blotting for 30 min. According to the protocol of the Dynabeads Protein G Immunoprecipitation Kit (#10007D, Invitrogen), 50 μL Dynabeads and 2 μg of a primary antibody were added, and the mixture was transferred to 4 °C for 3 h. Then, 1 g of cell lysate protein was added to the mixture, mixed, and moved to 4 °C for incubation overnight on a rotator. The mixture was centrifuged and the pellet was washed gently with PBS for 3 times. The mixture of bound protein to antibody and Dynabead was resuspended with 20 μL elution buffer and 5 μL 5× protein loading buffer and heated at 70 °C for 10 min to free and denature the protein. The tube was placed in a magnetic frame to separate the beads from the eluted protein in the supernatant, which was collected for SDS PAGE electrophoresis.

### Protein half-life assays

Cells were treated with cycloheximide (CHX) (MedChemExpress, Monmouth Junction, NJ, USA) at 100 μg/mL to inhibit protein synthesis and harvested at various time points (0, 1, 2, and 3 h). Cell proteins were extracted as described for Western blotting analysis. Protein levels of FBXO22 and p57^Kip2^ over the treatment duration were measured by Western blotting analysis.

### Proteasome inhibition

Cells were treated with 20 μM MG132, a proteasome inhibitor (MedChemExpress, NJ, USA) or DMSO (Sigma-Aldrich, MO, USA) for 6 h and harvested for proteins extraction. Protein levels of FBXO22 and p57 were detected by Western blotting analysis.

### Ubiquitination experiment

CC cells with altered FBXO22 expression were co-transfected with an ubiquitin expression vector. Cells were treated with 20 μM MG132 for 6 h and lysed with 10 μM N-ethylmaleimide (Sigma-Aldrich, MO, USA) on ice for 30 min. Total protein was extracted and its concentration was determined with BCA kit. p57^Kip2^ was immunoprecipitated as described in the IP assay above. Ubiquitinated p57^Kip2^ was detect3ed with an ubiquitin antibody (1:500, sc-166355, Santa Cruz) by Western blotting analysis.

### Tumor xenograft experiment

Five-week-old female nude mice were randomly separated into 4 different groups for subcutaneous transplantation of SiHa cells stably transfected with FBXO22 shRNA or cDNA expression vectors and their corresponding control vectors (2 × 10^6^ cells were injected per mouse). The size of xenograft tumors was measured twice a week with a caliper in two dimensions. The volume of xenograft tumors was calculated according to the following formula: Volume = *L* × *W*^2^/2 (*L* and *W*, respectively, represented the maximum diameter and shortest diameter of the tumor tissues). The mice were euthanized after six weeks and the tumors were dissected and weighted.

### Patients and tissue specimens

A tissue microarray, including 116 cases of CC tissues and 31 adjacent tissues with their clinical and prognosis data, was obtained from Shanghai Outdo Biotech Company (Shanghai, China). Furthermore, 10 paired fresh specimens of CC tissues and adjacent non-tumor tissues obtained from the Second Affiliated Hospital of Wenzhou Medical University were analyzed for Western blotting analysis.

### Statistical analysis

All data are presented as the means ± SEM. Chi-square test was used to analyze the positive rate of FBXO22 protein in various cervical tissues, and the correlation between the expression of FBXO22 protein in patients with CC and clinicopathological data. Overall survival (OS) and disease-free survival (DFS) were analyzed by Kaplan–Meier survival analysis and Log-rank statistical test. Univariate analysis and Cox multivariate survival regression analysis were performed to establish the independent prognostic factors for DFS and OS. Hazard ratio (HR) with a 95% confidence interval (CI) was used to represent relative risk in Cos multivariate survival analysis. Student’s *t*-test was utilized to analyze the significant differences between two groups, while ANOVA was used to examine statistical differences among multiple groups. Multiple *t*-tests were used to compare the data of two growth curves at different time points, and two-factor analysis of variance was used to compare the data of multiple survival curves at different time points. *P* < 0.05 was considered significant. GraphPad 6.0 and SPSS 25.0 statistical software were used to plot and analyze the significant differences among the treatment groups, respectively.

## Supplementary information

Supplementary table 1

Supplementary table 2

Supplementary table 3

Supplementary figures

Original WB Image

aj-checklist

## Data Availability

The datasets generated in the current study are available from the corresponding authors on reasonable request.
