# Supplementary material for: Fbxo22 promotes cervical cancer progression via targeting p57Kip2 for ubiquitination and degradation
Source: Cell Death Dis. 2022 Sep 20;13(9):805. doi: 10.1038/s41419-022-05248-z (PMC9489770; doi:10.1038/s41419-022-05248-z)
Supplement: Supplementary file 1 — Supplementary table 1 [file 41419_2022_5248_MOESM1_ESM.docx]

**Supplementary Table 1. Relationship between FBXO22 expression and clinicopathological parameters in 116 cases cervical cancer patients.**

| Features | Total  (n=116) | FBXO22 expression | | High rate (%) | *P* Value |
| --- | --- | --- | --- | --- | --- |
|  |  | high | low |  |  |
| **Age（years）** |  |  |  |  | 0.704 |
| ≤ 45 | 58 | 36 | 22 | 62.07 |  |
| > 45 | 58 | 34 | 24 | 58.62 |  |
| **Pathologic type** |  |  |  |  | 0.475 |
| SCC | 108 | 64 | 44 | 59.26 |  |
| AD/ASC | 8 | 6 | 2 | 75.00 |  |
| **Histology Grade** |  |  |  |  | 0.015 |
| I/II | 14 | 7 | 7 | 50.00 |  |
| III | 83 | 69 | 14 | 83.10 |  |
| Null | 18 |  |  |  |  |
| **Lymph node** |  |  |  |  | 0.033 |
| No | 95 | 53 | 42 | 55.79 |  |
| Yes | 21 | 17 | 4 | 80.95 |  |
| **TNM stage** |  |  |  |  | 0.071 |
| I-II | 94 | 53 | 41 | 56.38 |  |
| III-IV | 22 | 17 | 5 | 77.27 |  |
| **HPV infection** |  |  |  |  | 0.895 |
| Positive | 89 | 55 | 34 | 61.80 |  |
| Negative | 15 | 9 | 4 | 60.00 |  |
| Null | 12 |  |  |  |  |

**Note:** SCC: squamous cell carcinoma, AD: adenocarcinoma, ASC: Adenosquamous carcinoma.
