# Supplementary material for: Fbxo22 promotes cervical cancer progression via targeting p57Kip2 for ubiquitination and degradation
Source: Cell Death Dis. 2022 Sep 20;13(9):805. doi: 10.1038/s41419-022-05248-z (PMC9489770; doi:10.1038/s41419-022-05248-z)
Supplement: Supplementary file 2 — Supplementary table 2 [file 41419_2022_5248_MOESM2_ESM.docx]

**Supplementary Table 2. Univariate analysis and multivariate Cox stepwise regression analysis of the clinicopathologic factor with overall survival in patients with cervical cancer.**

| **variables** | **Univariate analysis** | | |  | **Multivariate analysis** | | |  |
| --- | --- | --- | --- | --- | --- | --- | --- | --- |
|  | HR | 95%CI | *p* value |  | HR | 95%CI | *p* value |  |
| FBXO22 | 4.122 | 1.586-10.709 | 0.004 |  | 4.448 | 1.692-11.694 | 0.002 |  |
| Age | 6.985 | 2.684-18.174 | <0.001 |  | 8.248 | 3.128-21.752 | <0.001 |  |
| Grade | 2.386 | 0.891-6.394 | 0.084 |  |  |  |  |  |
| TNM stage | 4.696 | 2.327-9.480 | <0.001 |  | 3.986 | 1.936-8.206 | <0.001 |  |
| type | 1.748 | 0.532-5.740 | 0.358 |  |  |  |  |  |
| HPV | 0.978 | 0.337-2.838 | 0.967 |  |  |  |  |  |
