# Supplementary material for: Fbxo22 promotes cervical cancer progression via targeting p57Kip2 for ubiquitination and degradation
Source: Cell Death Dis. 2022 Sep 20;13(9):805. doi: 10.1038/s41419-022-05248-z (PMC9489770; doi:10.1038/s41419-022-05248-z)
Supplement: Supplementary file 3 — Supplementary table 3 [file 41419_2022_5248_MOESM3_ESM.docx]

**Supplementary Table 3. Univariate analysis and multivariate Cox stepwise regression analysis of the clinicopathologic factor with disease free survival in patients with cervical cancer.**

| **variables** | **Univariate analysis** | | |  | **Multivariate analysis** | | |  |
| --- | --- | --- | --- | --- | --- | --- | --- | --- |
|  | HR | 95%CI | *p* value |  | HR | 95%CI | *p* value |  |
| FBXO22 | 3.887 | 1.617–9.344 | 0.002 |  | 4.135 | 1.703–10.036 | 0.002 |  |
| Age | 4.617 | 2.100-10.151 | <0.001 |  | 5.401 | 2.431–12.003 | <0.001 |  |
| Grade | 1.783 | 0.789–4.031 | 0.164 |  |  |  |  |  |
| TNM stage | 3.897 | 1.987–7.461 | <0.001 |  | 3.348 | 1.686–6.647 | 0.001 |  |
| type | 2.259 | 0.798–6.396 | 0125 |  |  |  |  |  |
| HPV | 1.179 | 0.412–3.380 | 0.759 |  |  |  |  |  |
