# Supplementary material for: Fbxo22 promotes cervical cancer progression via targeting p57Kip2 for ubiquitination and degradation
Source: Cell Death Dis. 2022 Sep 20;13(9):805. doi: 10.1038/s41419-022-05248-z (PMC9489770; doi:10.1038/s41419-022-05248-z)
Supplement: Supplementary file 4 — Supplementary figures [file 41419_2022_5248_MOESM4_ESM.pdf]

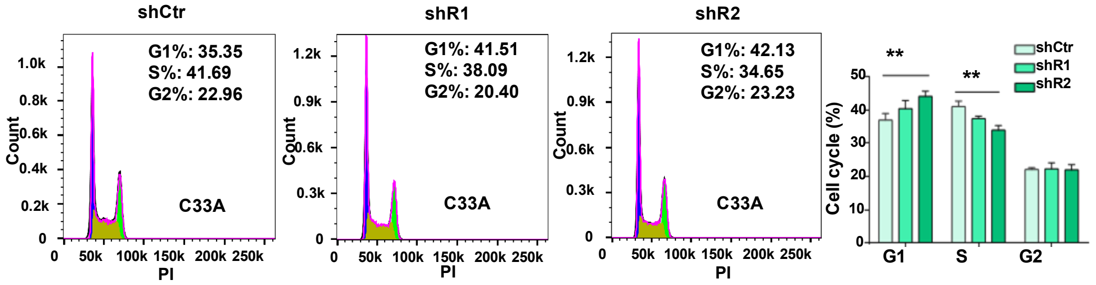

Supplementary figure 1. Knockdown of FBXO22 inhibits cell cycle progression from G1 to S phase in C33A cells.

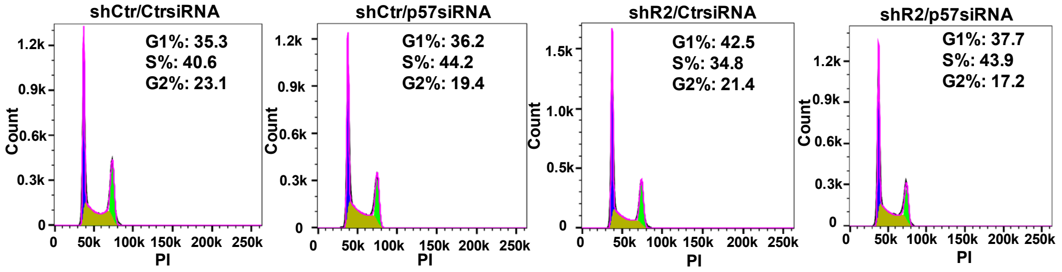

Supplementary figure 2. Representative images of cell cycle in C33A cells after transfection with FBXO22 shRNA2 and p57 siRNA.

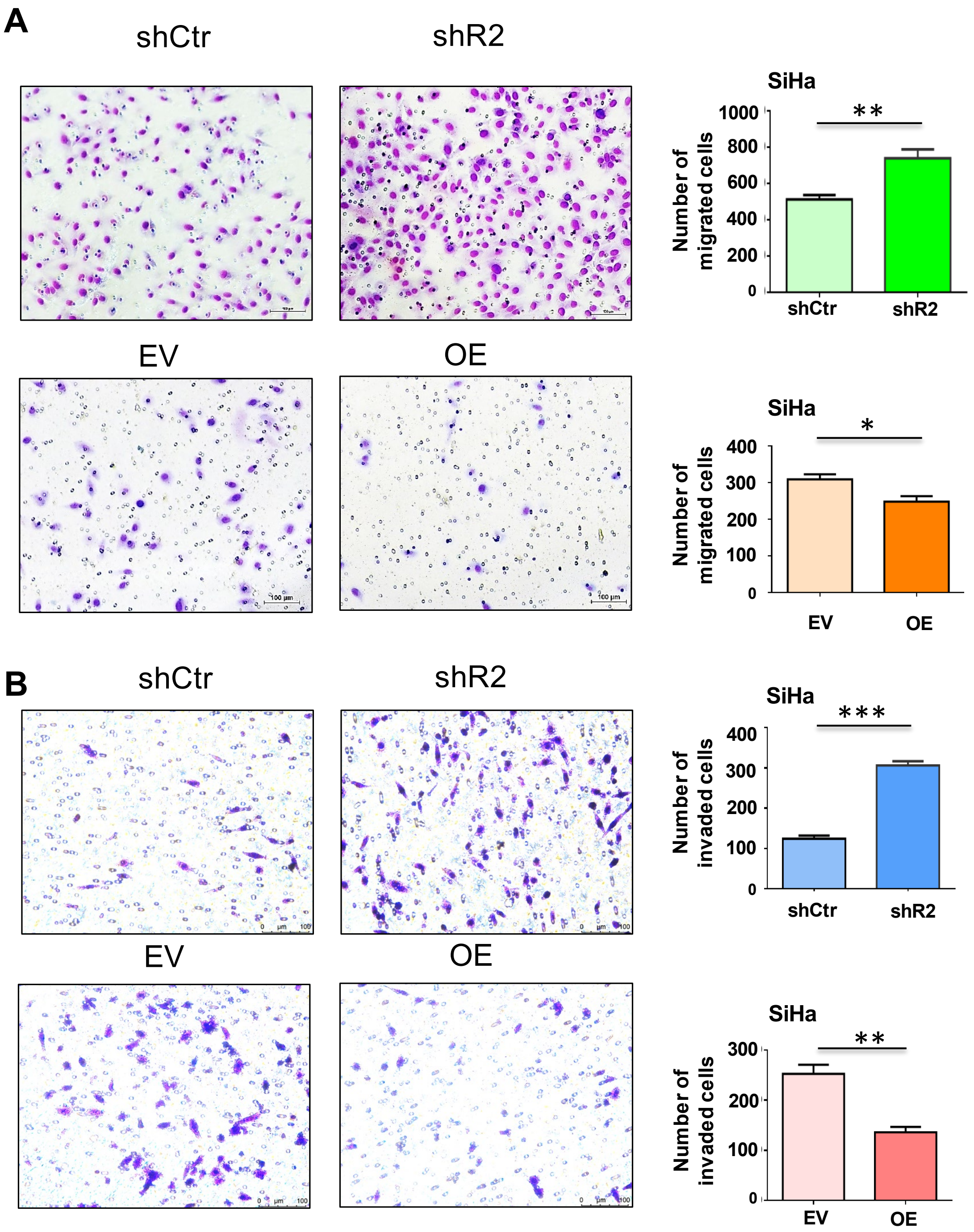

Supplementary figure 3. FBXO22 inhibits cell migration and invasion in SiHa cells. A-B Migration (A) and invasion (B) was determined after transfection with with FBXO22 control cells (shCtr), FBXO22 knockdown cells (shR2), SiHa control cells (EV), and FBXO22 overexpressing cells (OE). Each data point represents the mean ± SEM from triplicate measurements. * $P < 0.05$, ** $P < 0.01$, *** $P < 0.001$.

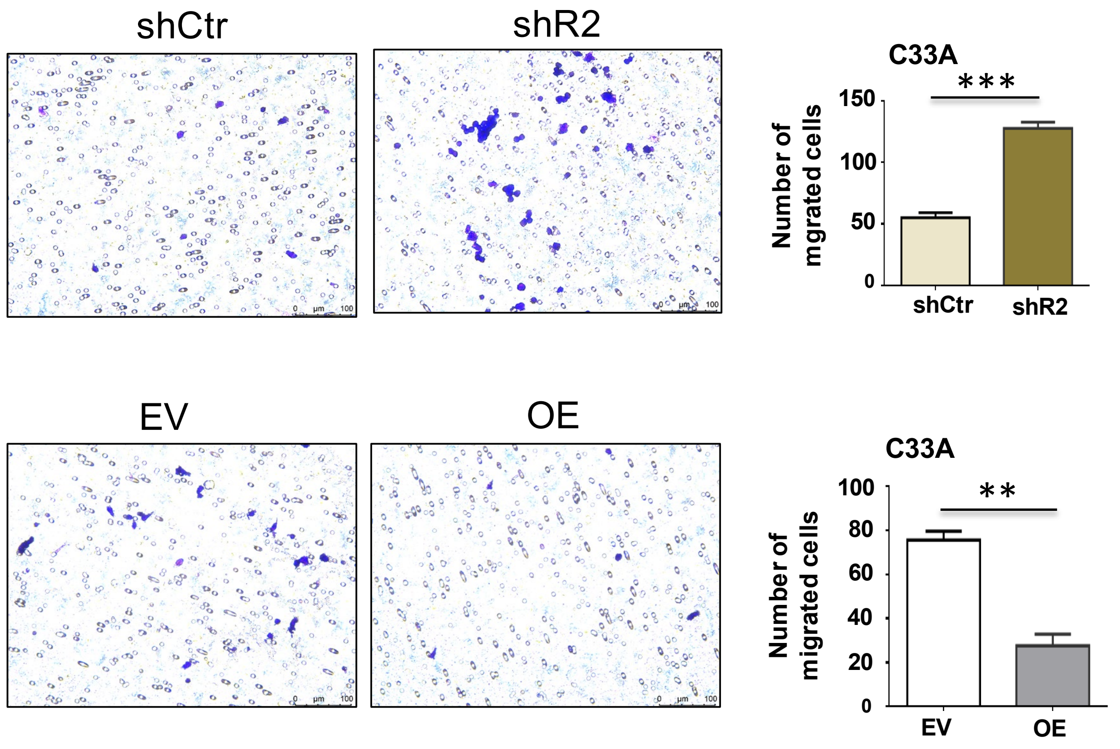

Supplementary figure 4. FBXO22 inhibits cell migration in C33A cells.

Migration was determined after transfection with FBXO22 control cells (shCtr), FBXO22 knockdown cells (shR2), SiHa control cells (EV), and FBXO22 overexpressing cells (OE). Each data point represents the mean ± SEM from triplicate measurements. ** $P < 0.01$, *** $P < 0.001$.

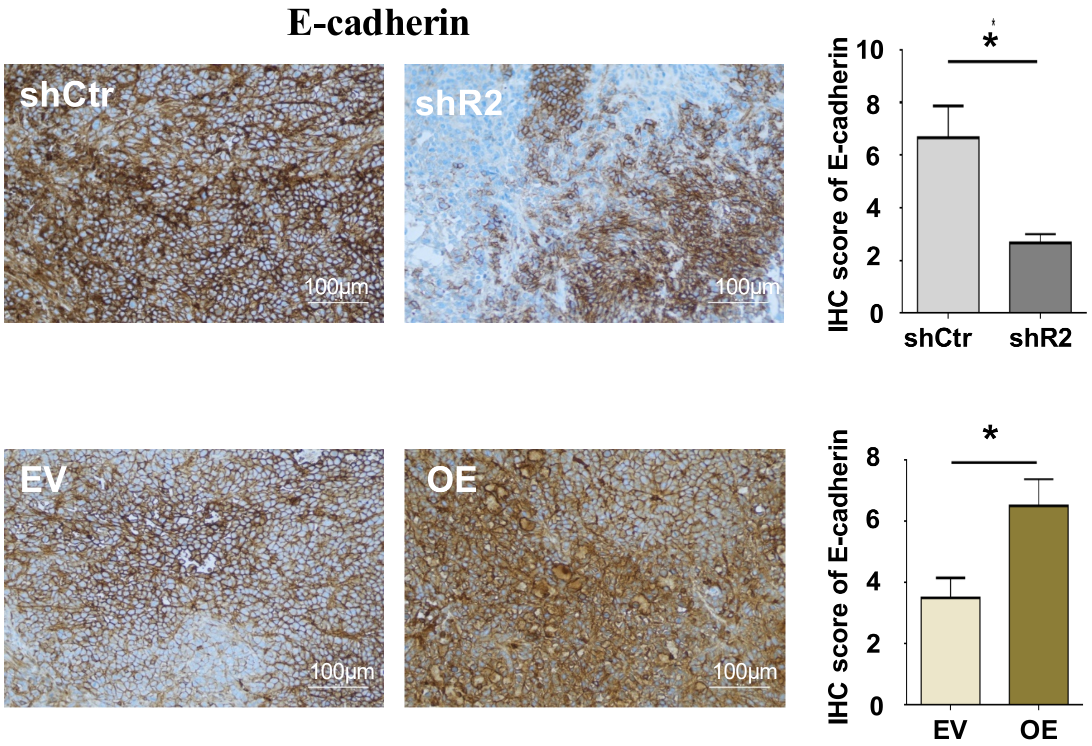

Supplementary figure 5. EMT in the xerographs tumors derived from SiHa control cells (shCtr), FBXO22 knockdown cells (shR2), SiHa control cells (EV), and FBXO22 overexpressing cells (OE).

Representative images of IHC staining with anti-E-cadherin antibody in tissue sections of the tumors formed by the control, FBXO22 knockdown (shR2), or overexpressing (OE) SiHa cells (200×, magnification). **P* < 0.05, Data are expressed as mean ± SEM.

A

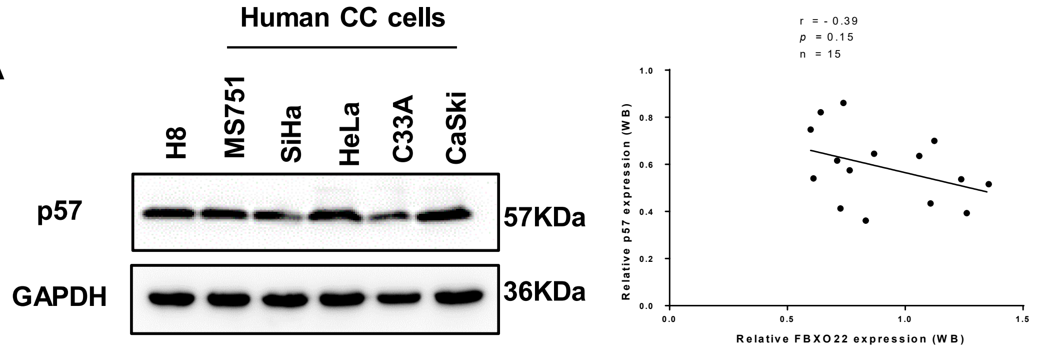

Supplementary figure 6. A, The relative protein levels of p57kip2 in one human immortalized cervical cell line (H8) and five various human CC cell lines by Western blotting method. B, Linear correlation analysis of FBXO22 and p57Kip2 in five various human CC cell lines.

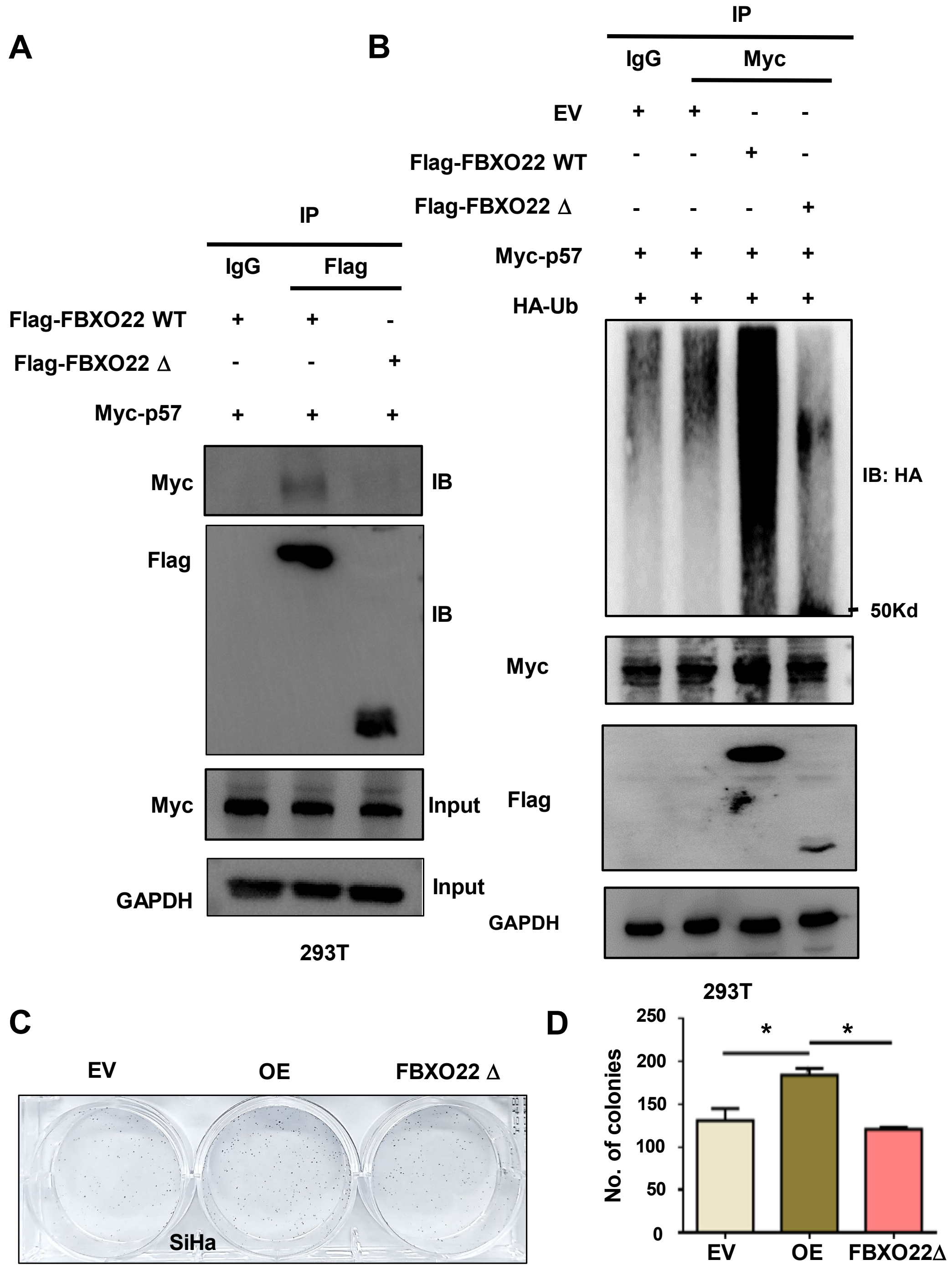

Supplementary figure 7. A, 293T cells were transfected with myc-p57, Flag-FBXO22 and Flag-FBXO22Δ. FBXO22 and p57Kip2 proteins physically interact with each other in 293T cells by IP assays as detected by immunoblotting. B, The levels of ubiquitinated ectopic p57Kip2 was measured in immunoprecipitated p57Kip2 in the CC cells with FBXO22 control (EV), overexpression (Flag-FBXO22) or FBXO22 mutation (FBXO22Δ). 293T cells were transfected with myc-p57, HA-ubiquitin, Flag-FBXO22 and Flag-FBXO22Δ. C-D. Overexpressed FBXO22 promoted colony formation of SiHa cells. Each data point is means ± SEM from triplicate measurements. * $P < 0.05$.

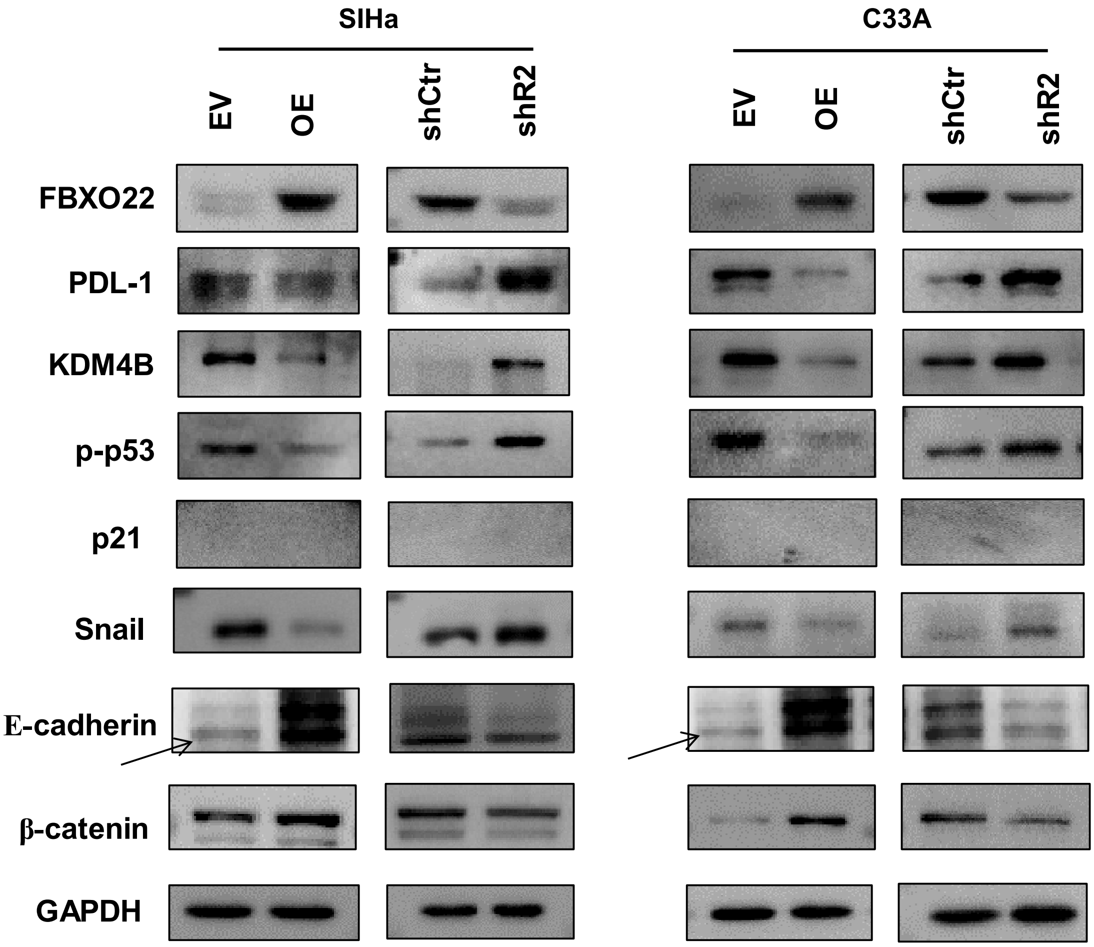

Supplementary figure 8: FBOX22 targets previously identified proteins and several key proteins in cervical cancer.

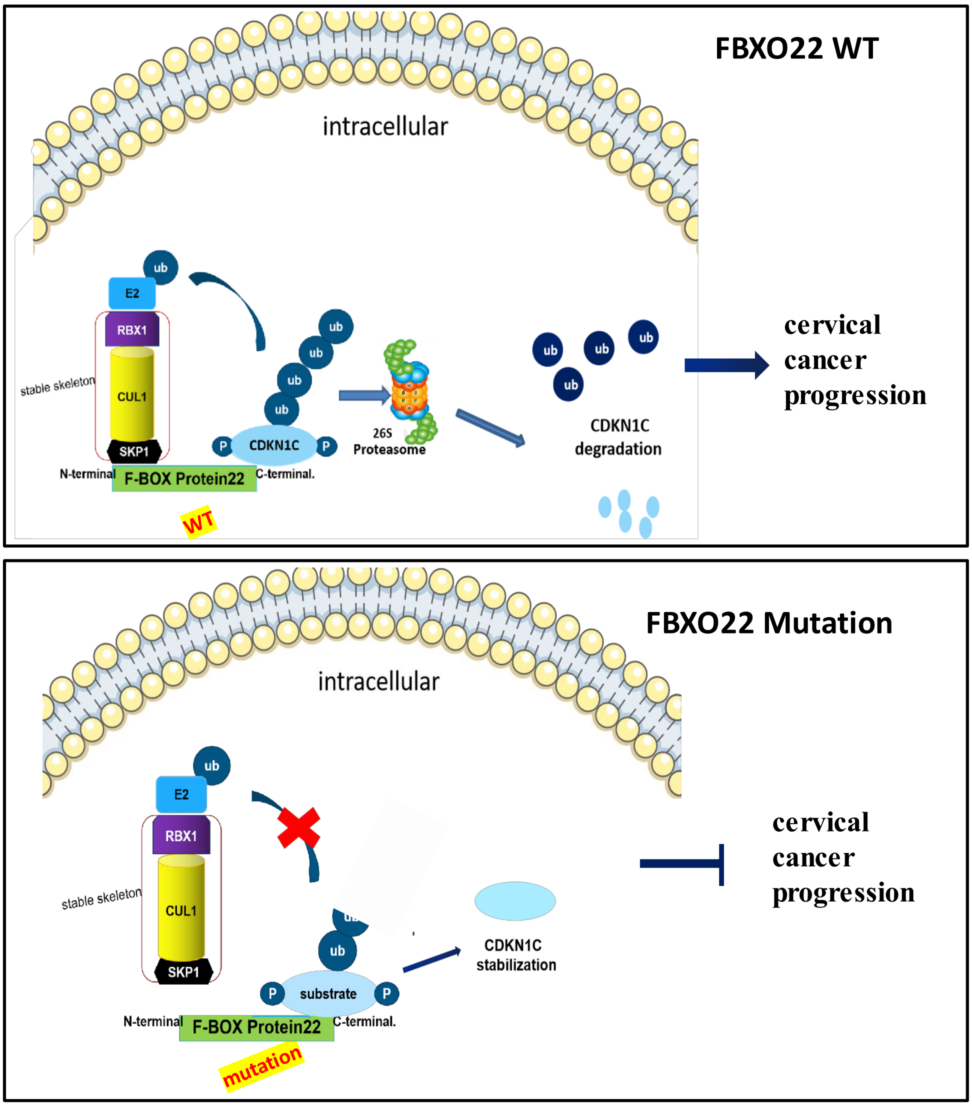

Supplementary figure 9: The schematic view of the proposed mechanism underlying the effect of FBXO22 and its interaction with p57kip2 in cervical cancer.
