# Supplementary material for: Fbxo22 promotes cervical cancer progression via targeting p57Kip2 for ubiquitination and degradation
Source: Cell Death Dis. 2022 Sep 20;13(9):805. doi: 10.1038/s41419-022-05248-z (PMC9489770; doi:10.1038/s41419-022-05248-z)

Fig. 1B

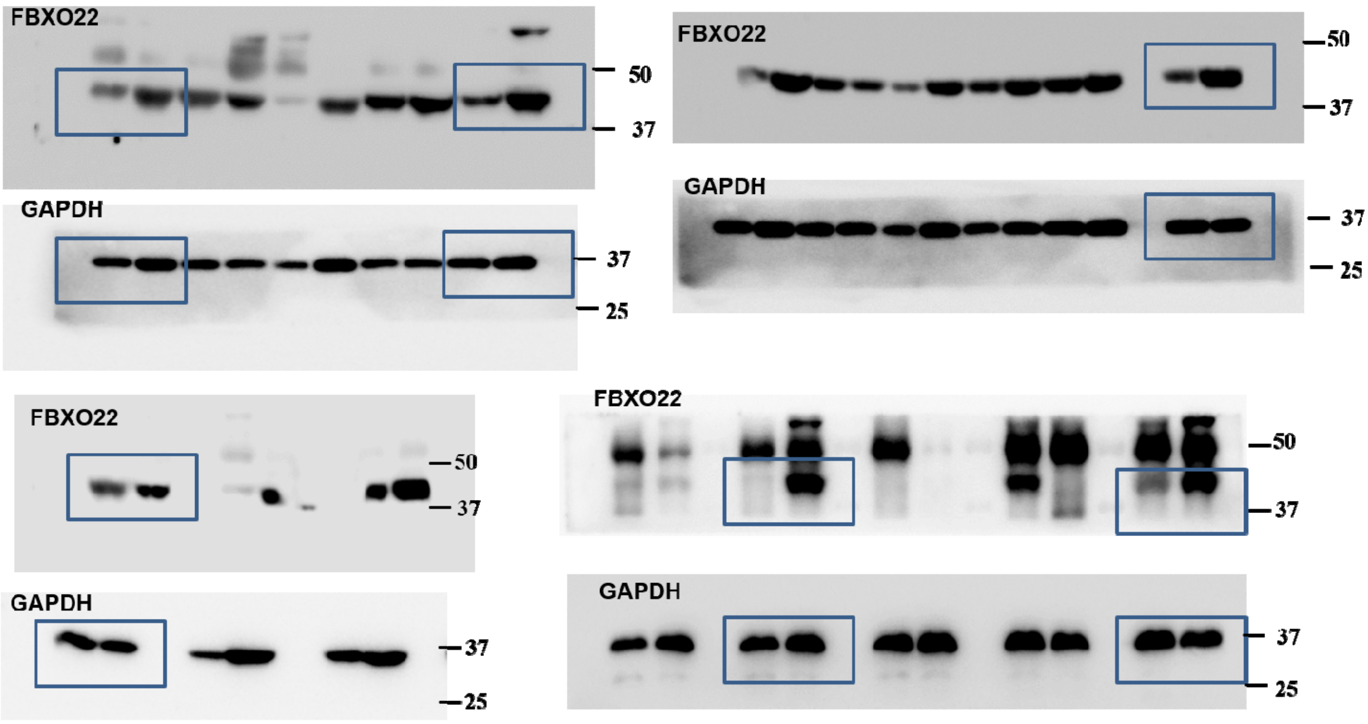

Fig. 2B

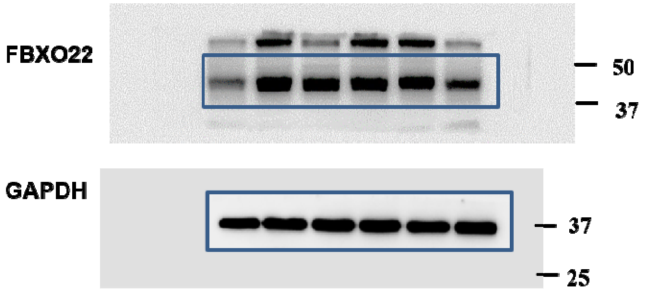

Fig. 2D

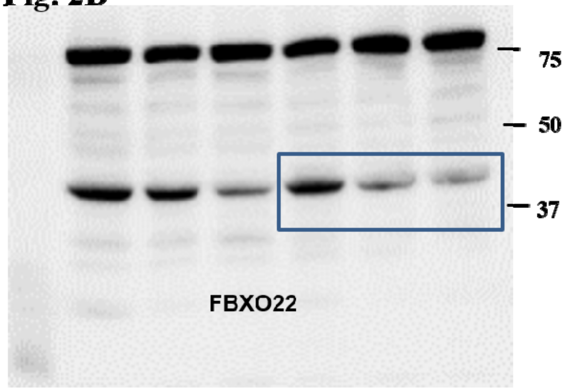

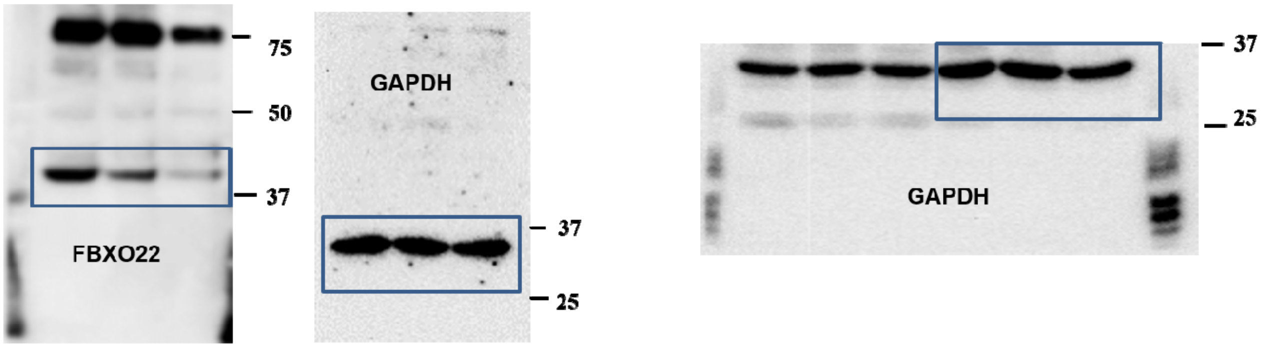

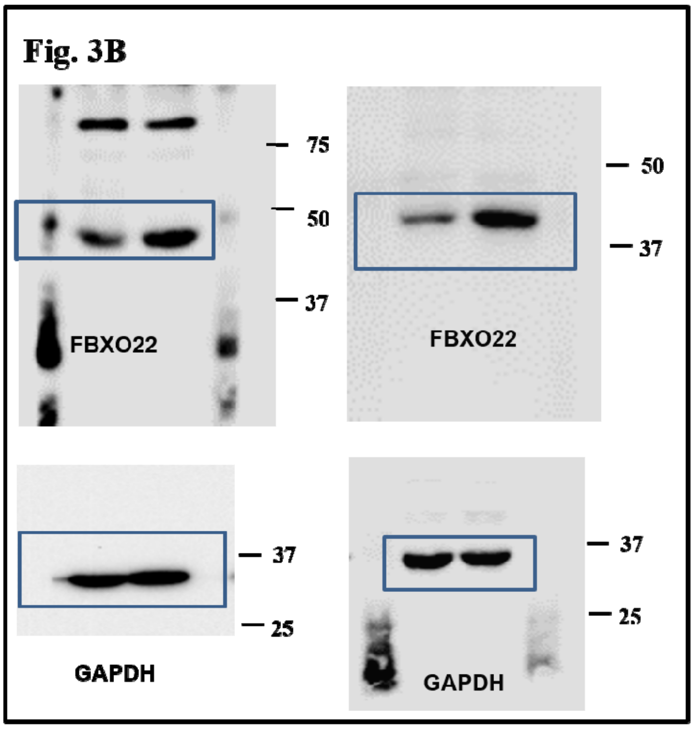
Fig. 3B
75
50
37
FBXO22
50
37
FBXO22
37
25
GAPDH
37
25
GAPDH

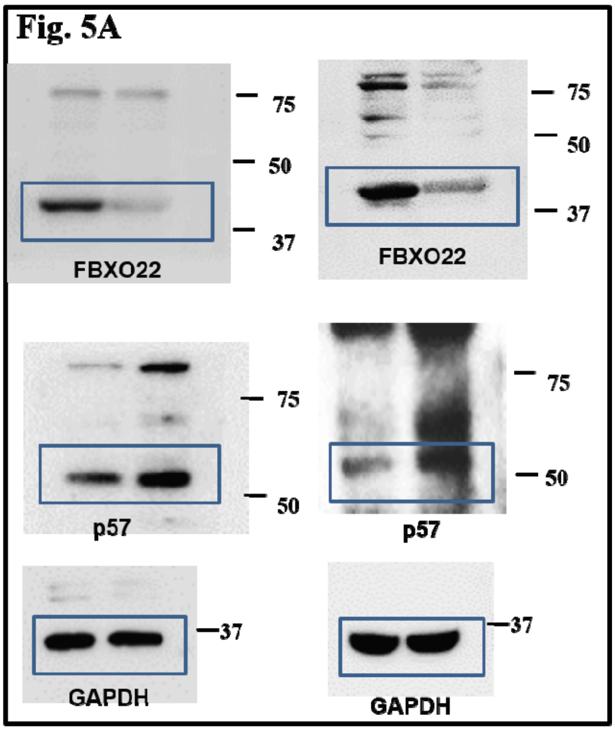
Fig. 5A
75
50
37
FBXO22
75
50
37
FBXO22
75
50
p57
75
50
p57
37
GAPDH
37
GAPDH

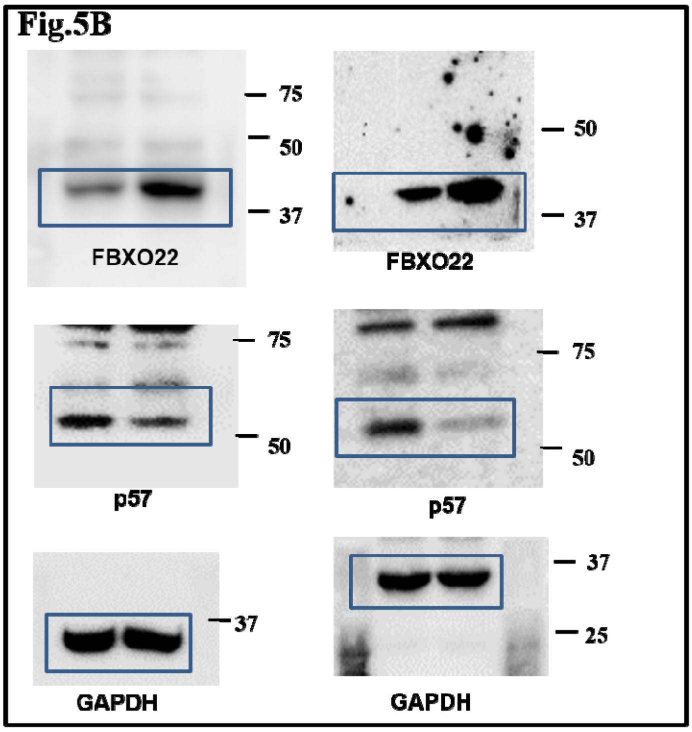
Fig.5B
75
50
37
FBXO22
50
37
FBXO22
75
50
p57
75
50
p57
37
GAPDH
37
25
GAPDH

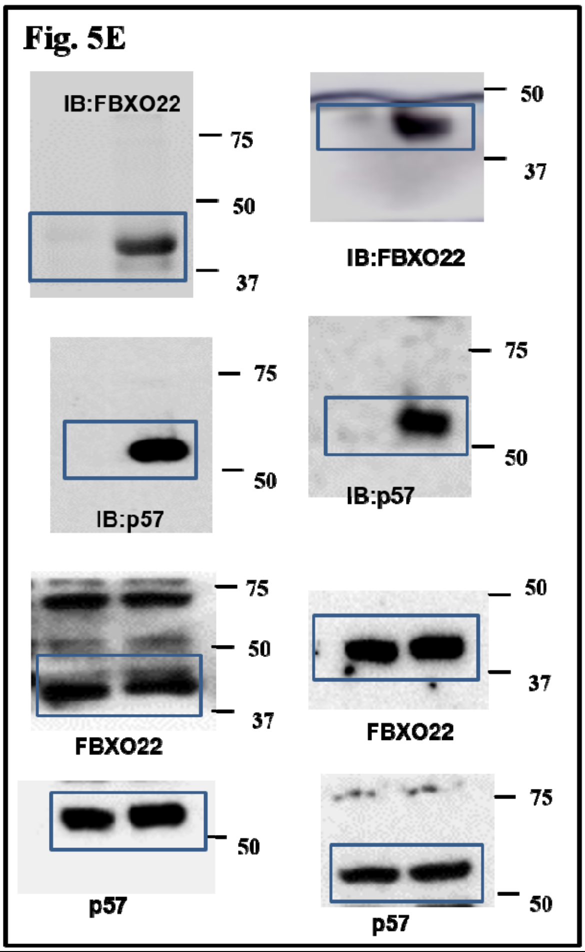
Fig. 5E
IB:FBXO22
75
50
37
50
37
IB:FBXO22
75
50
IB:p57
75
50
IB:p57
75
50
37
FBXO22
50
37
FBXO22
50
p57
75
50
p57

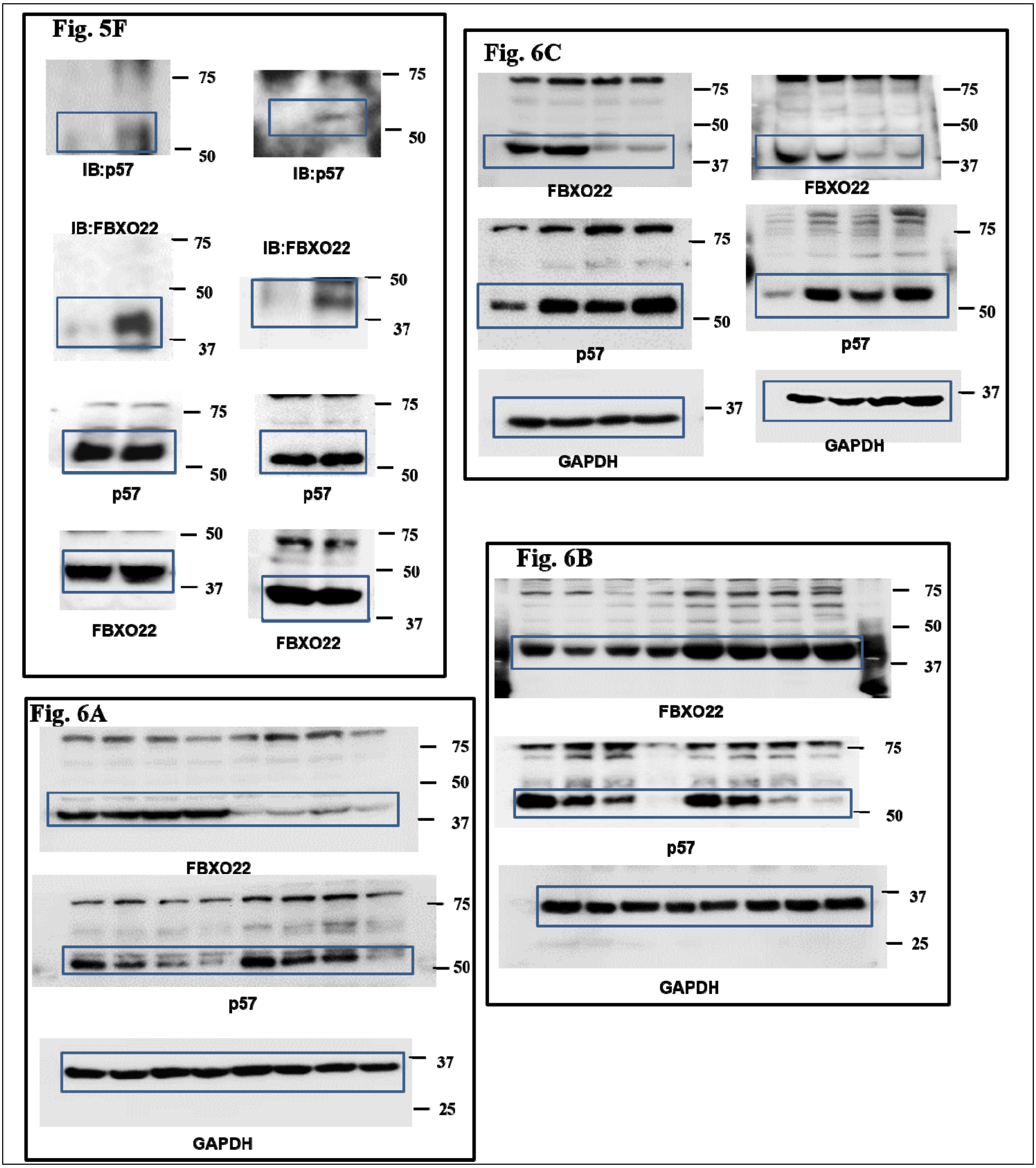

Fig. 5F
75
50
IB:p57
75
50
IB:p57
IB:FBXO22
75
50
37
IB:FBXO22
50
37
75
50
p57
75
50
p57
50
37
FBXO22
75
50
37
FBXO22
Fig. 6C
75
50
37
FBXO22
75
50
37
FBXO22
75
50
p57
75
50
p57
37
GAPDH
37
GAPDH
Fig. 6A
75
50
37
FBXO22
75
50
p57
37
25
GAPDH
Fig. 6B
75
50
37
FBXO22
75
50
p57
37
25
GAPDH

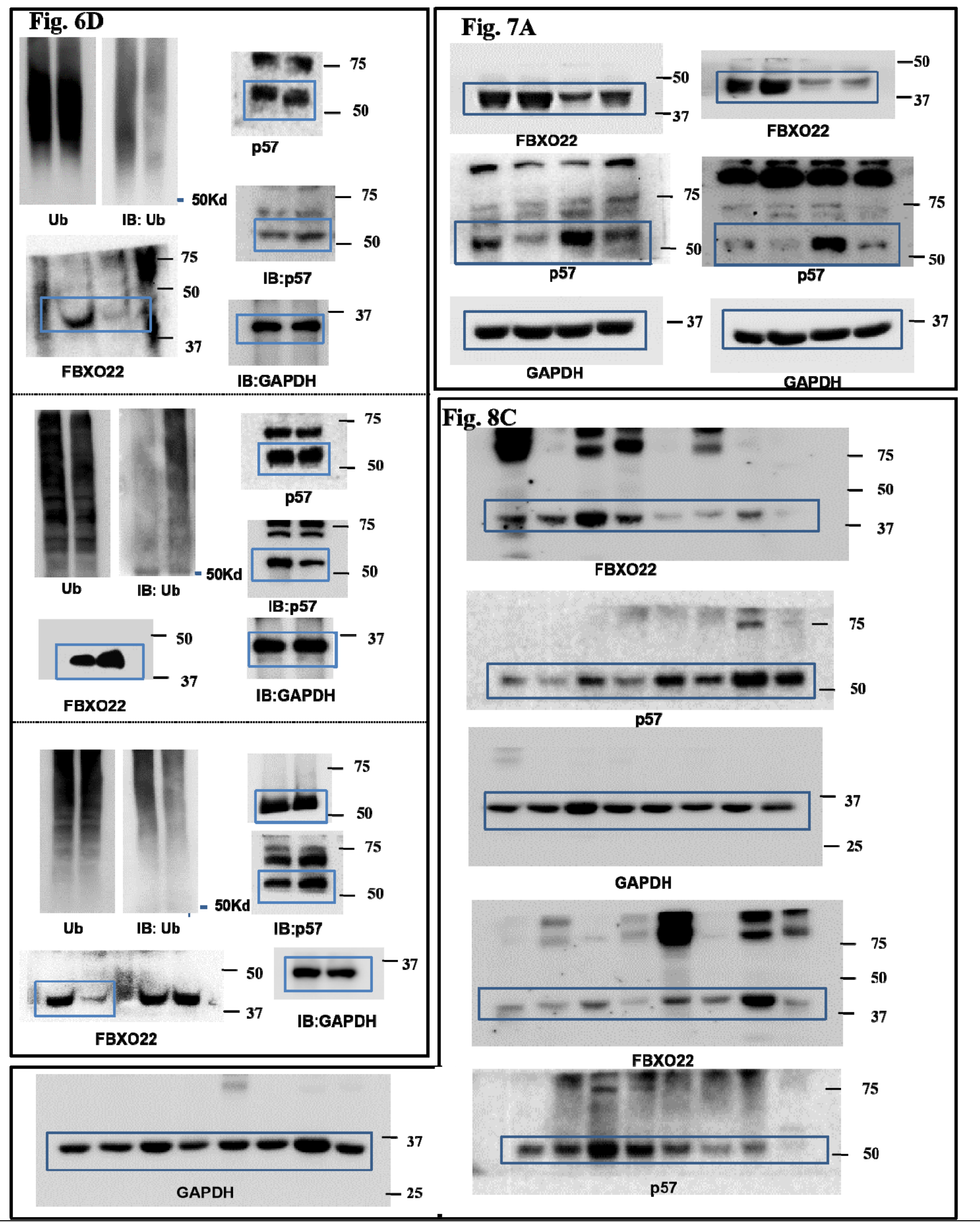
Fig. 6D
Ub
IB: Ub
50Kd
p57
IB:p57
FBXO22
IB:GAPDH
75
50
37
Fig. 7A
FBXO22
p57
GAPDH
Fig. 8C
FBXO22
p57
GAPDH
25

Fig. 8E

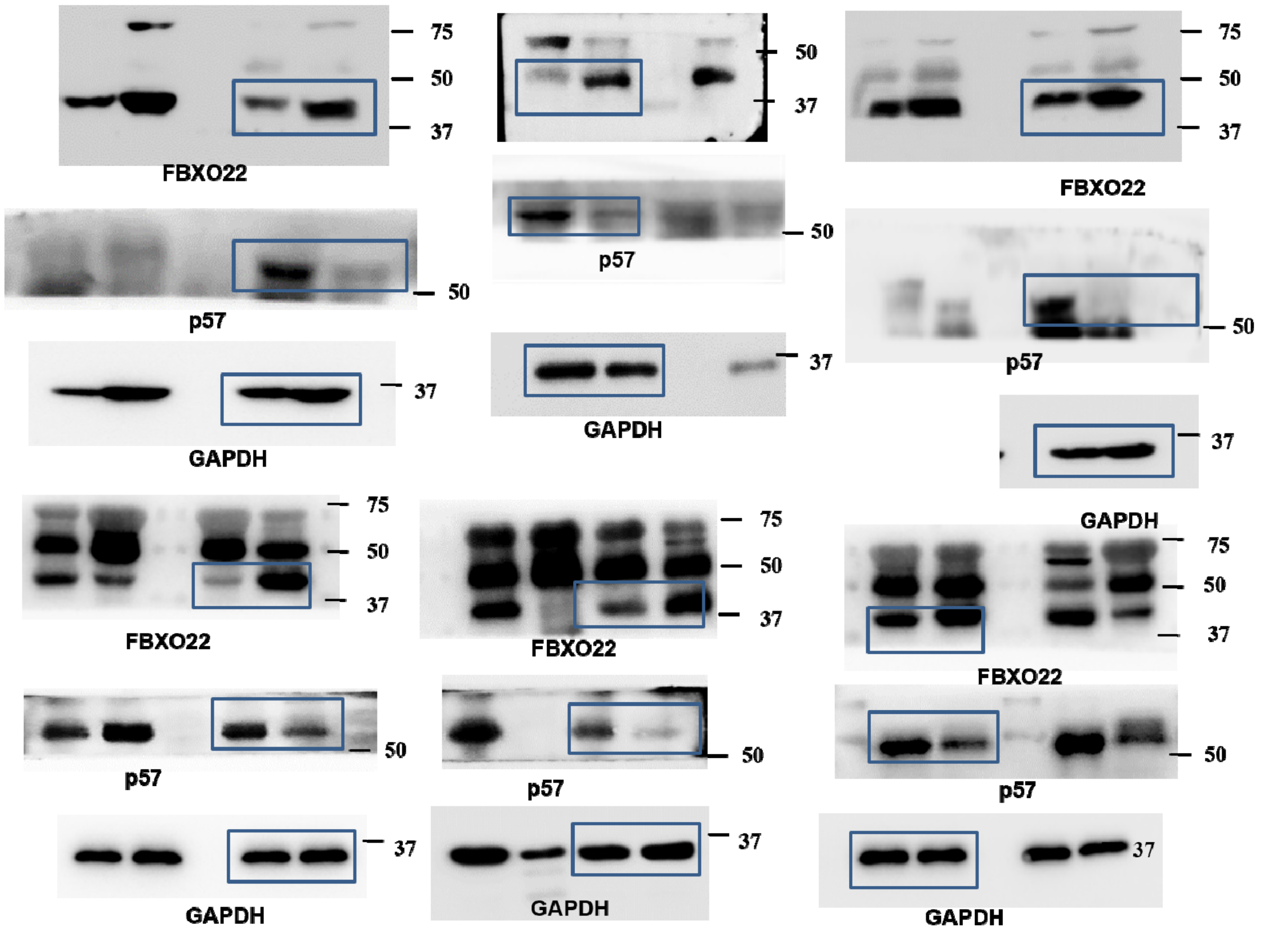

75
50
37
FBXO22
50
37
75
50
37
FBXO22
50
p57
50
p57
50
p57
37
GAPDH
37
GAPDH
37
GAPDH
75
50
37
FBXO22
75
50
37
FBXO22
75
50
37
FBXO22
50
p57
50
p57
50
p57
37
GAPDH
37
GAPDH
37
GAPDH

Supplementary Fig. 6A

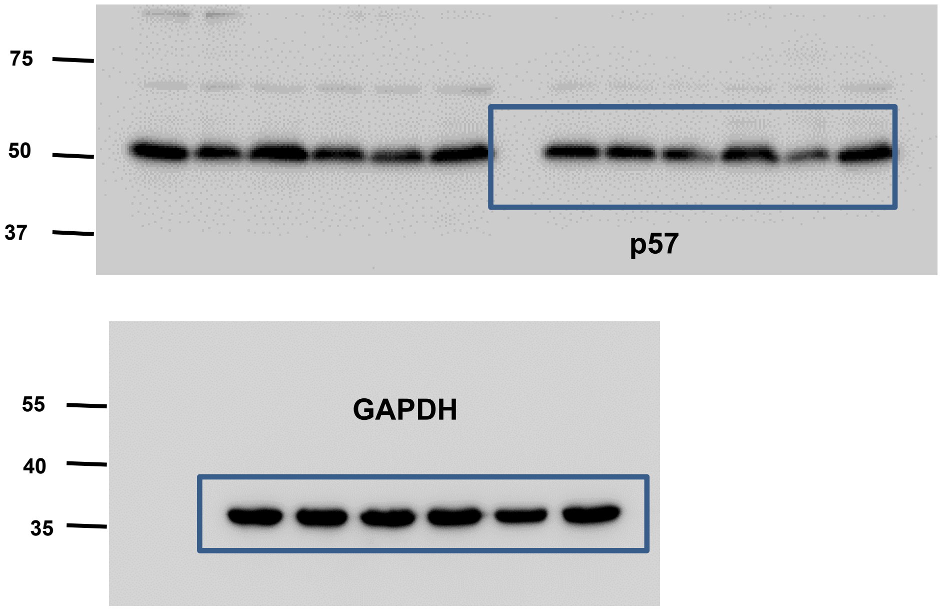

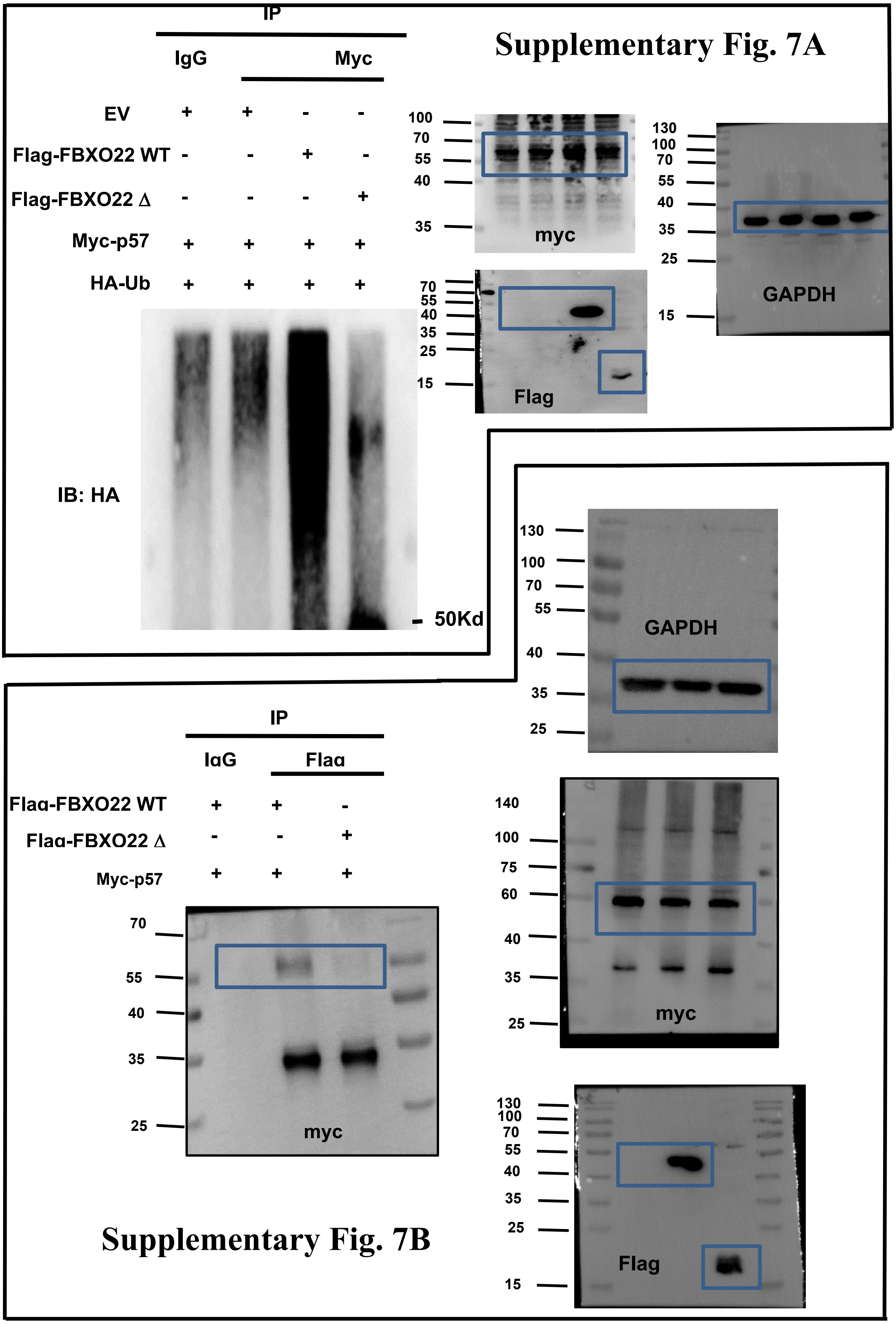

Supplementary Fig. 7A

Supplementary Fig. 7B

Supplementary Fig. 8

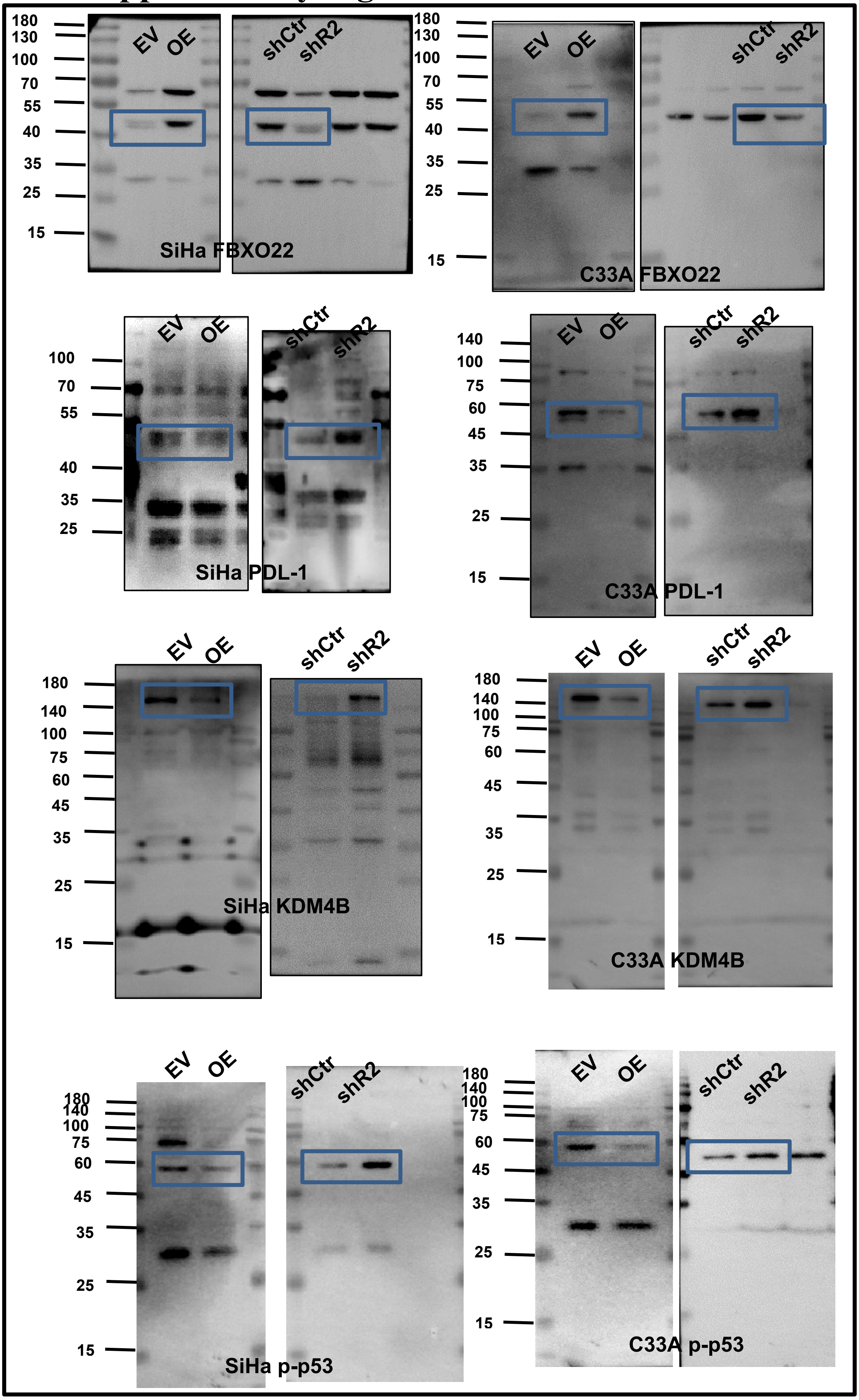

Supplementary Fig. 8

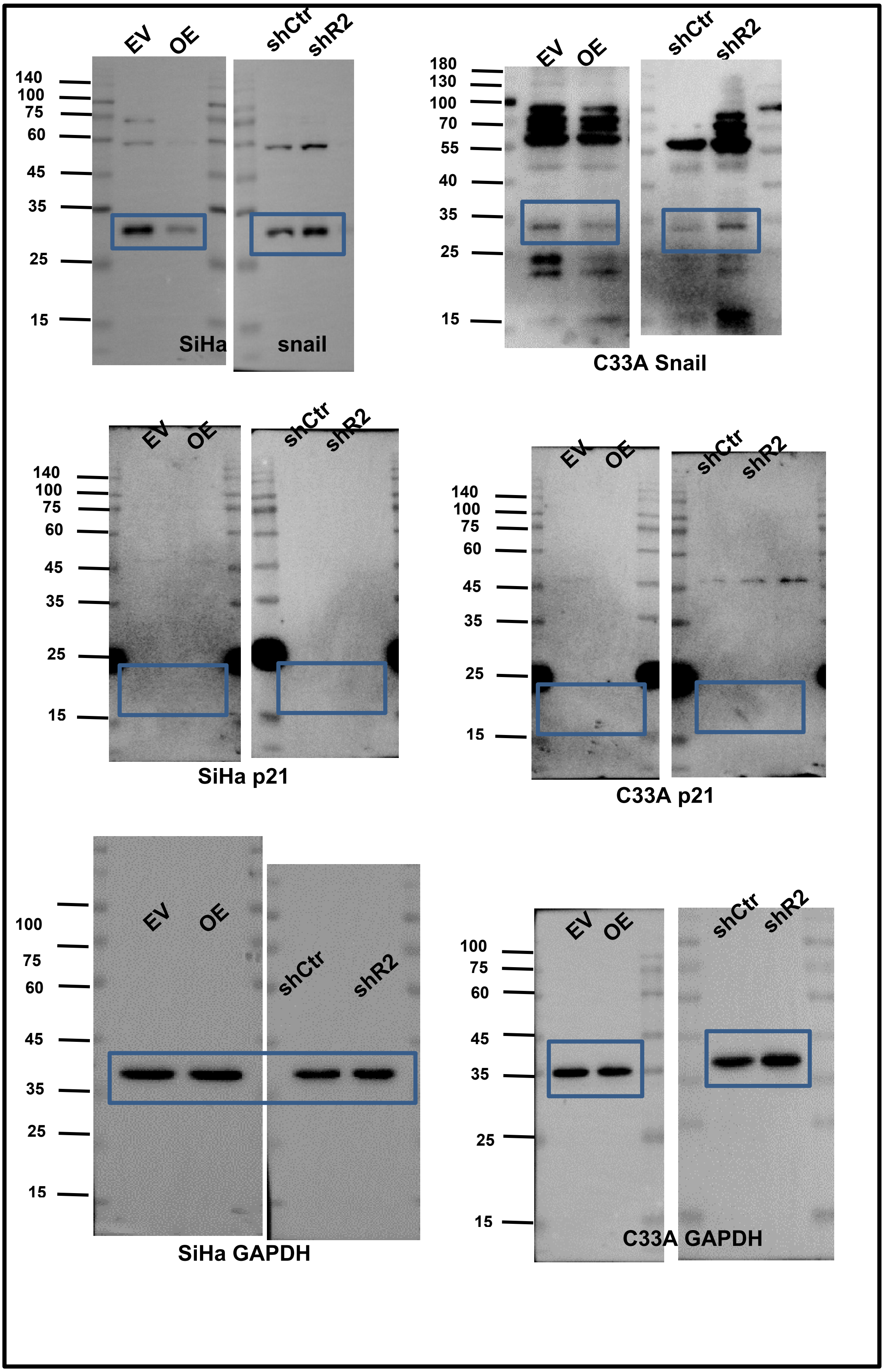

Supplementary Fig. 8

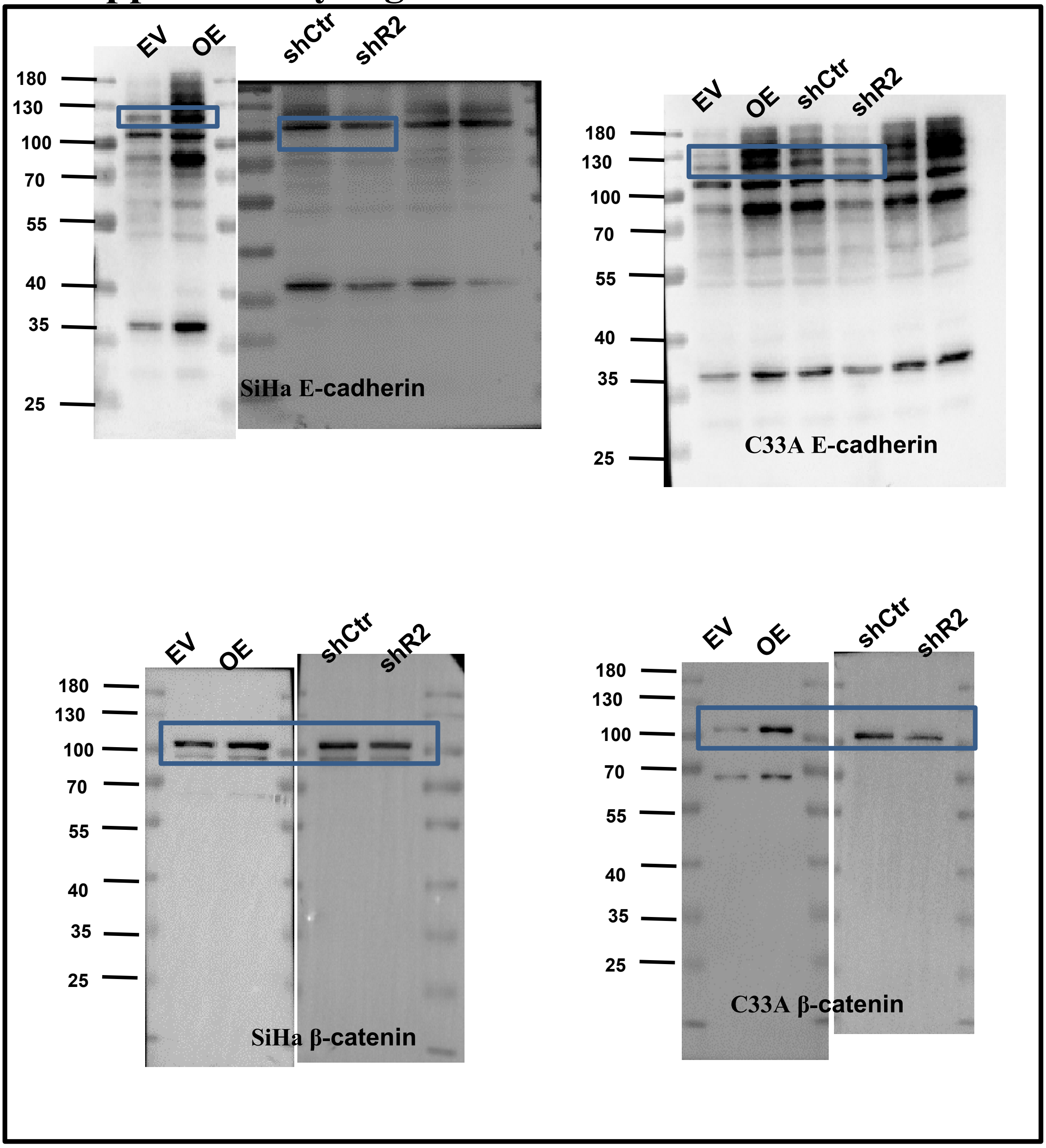

Supplement: Supplementary file 5 — Original WB Image [file 41419_2022_5248_MOESM5_ESM.pdf]
